# The effect of similarity between owner’s values and their perceptions of their pet’s values on life satisfaction

**DOI:** 10.3389/fpsyg.2022.1029883

**Published:** 2022-10-28

**Authors:** Joanne Sneddon, Sheng Ye, Julie A. Lee

**Affiliations:** ^1^Centre for Human and Cultural Values, Business School, University of Western Australia, Crawley, WA, Australia; ^2^Business Administration, Business School, East China University of Science and Technology, Shanghai, China

**Keywords:** personal values, life satisfaction, pets, value similarity, congruence, response surface analysis

## Abstract

It is often assumed that pet ownership improves peoples’ wellbeing, but evidence of this pet effect has been mixed. We extended past research on pet personality, the pet effect, and value congruence to examine whether people perceive their pets to have humanlike values and if owner-pet values similarity has a positive effect on owners’ life satisfaction. In a large and diverse sample of Australian dog and cat owners, we find that people imbue their dogs and cats with humanlike values in a way that reflects the theoretical circular structure of values. Importantly, perceptions of the values of dogs and cats differed in that dogs were perceived to prioritize more social-focus values, whereas cats were perceived to prioritize more personal-focus values. Additionally, we find that similarity in the values profile of dog owners and their dogs is positively associated with life satisfaction, but this was not the case for cats. However, when we examined associations between individual values similarity and life satisfaction, our results suggest a more complex and nuanced picture of both direct and indirect similarity effects.

## Introduction

Similarities in values between individuals have been associated with wellbeing across a range of relationship contexts. For instance, greater similarity between personal values and the values of fellow citizens (e.g., Hanel et al., 2020; Wolf et al., 2021), community members (e.g., Sortheix et al., 2013), student peers (e.g., Sortheix and Lönnqvist, 2015), classmates (e.g., Benish-Weisman et al., 2020), and romantic partners (e.g., Leikas et al., 2018) has been positively associated with aspects of wellbeing. While similarities in the values of close and distant others appear to be important predictors of a range of wellbeing outcomes, the role of values similarity in owner-pet relationships remains unexplored. This is a potentially fruitful avenue of research given the increasingly important role that pets play in human society and in the lives of their owners (Serpell and Paul, 2011; Kirk, 2019; Wells, 2019).

There has been considerable interest in the role that pet ownership plays in wellbeing, with some evidence to suggest that having a pet has positive wellbeing effects (e.g., Allen et al., 2001; McConnell et al., 2011; Bao and Schreer, 2016). However, the finding that simply having a pet improves wellbeing outcomes is not consistent across studies (reviewed in Friedmann and Son, 2009), suggesting that other factors may influence relations between pet ownership and wellbeing. For example, studies have shown greater wellbeing outcomes for owners who perceive their pets to have more socially supportive attributes (McConnell et al., 2011), view their pets as family members (McConnell et al., 2019), and give their pets support (Kanat-Maymon et al., 2021). There is also some evidence that greater similarity between the personality of owners and their perceptions of their pets is positively associated with wellbeing. For instance, similarity in owner-pet personality traits has been associated with greater life satisfaction (El-Alayli et al., 2006) and relationship satisfaction (Curb et al., 2013). It may be the case that when owners perceive their pets to have a similar personality, they feel a greater sense of social connection with them, resulting in more positive wellbeing outcomes (Epley et al., 2007; McConnell et al., 2011). In the current study, we extend existing research on values and on owner-pet similarity by examining whether owners perceive their pets to have humanlike values and whether similarities between owners’ values and perceptions of their pets’ values enhance wellbeing.

## Literature review

### Anthropomorphism and wellbeing

It is common for people to imbue their pets with humanlike characteristics (see Amiot and Bastian, 2015 for a review). People have been found to anthropomorphize their pets as loyal friends or even family members with whom they share a close emotional bond (e.g., Greenebaum, 2004; McConnell et al., 2017). The anthropomorphism of pets goes beyond the attribution of humanlike roles to include emotions and aspects of personality. For example, studies have shown that people imbue their pets with emotions such as happiness, anger, fear, and surprise (Martens et al., 2016; Arahori et al., 2017). In terms of personality, studies have shown that people imbue their pets with supportive traits (i.e., thoughtful, considerate, sympathetic; Epley et al., 2008; McConnell et al., 2019) and other traits (e.g., extraversion, agreeableness, neuroticism; Cavanaugh et al., 2008; Litchfield et al., 2017). People have even been found to have a pet-enhancement bias, in which they rated their pets more favorably on desirable personality traits than the average pet (El-Alayli et al., 2006). Further, imbuing pets with humanlike characteristics has been found to impact aspects of owner’s wellbeing. For instance, perceiving pets as family members has been positively associated with owner wellbeing (McConnell et al., 2019).

Research examining the effects of similarity between owners’ and perceptions of their pets’ personality on aspects of wellbeing has mixed results. For instance, owners’ ascription of supportive traits to their pet (e.g., thoughtful, considerate, and sympathetic) was associated with positive wellbeing outcomes, whereas other non-supportive traits (e.g., creativity, embarrassment) were not (McConnell et al., 2019). Curb et al. (2013) found a positive similarity effect on relationship satisfaction for only 4 out of 45 personality traits for dog owners and their perceptions of their dog (i.e., sharing possessions, enjoyment of running outside, ability to get along with peers, and even engagement in destructive activity). In contrast, Cavanaugh et al. (2008) found that dog owners reported higher relationship satisfaction when they perceived their dogs to have higher openness and agreeableness traits than themselves. However, little is known about whether the similarity between owners’ values and their perceptions of their pet’s values has an effect on owner’s subjective wellbeing in a similar way to that found in human relationships (e.g., Sortheix and Lönnqvist, 2015; Benish-Weisman et al., 2020; Wolf et al., 2021).

### Personal values

Personal values (e.g., power, benevolence) are defined as broad, motivational goals, that transcend situations and serve as guiding principles in people’s lives (Rokeach, 1973; Schwartz, 1992). Values are a central aspect of a person’s self-concept that reflect what is important to them in their life, they differ from other aspects of personality, such as traits, in various ways. First, personal values are seen as being inherently desirable and good (Roccas et al., 2014). Second, individuals order their values in a personal hierarchy of relative importance (Sagiv et al., 2017). For instance, for one person, kindness may be more important than achievement, but for another person, achievement may be more important than kindness. Third, because values are relatively broad, abstract goals, they transcend specific actions and situations and apply across contexts (Schwartz, 2012). Fourth, values tend to be stable in terms of their relative importance throughout adulthood (e.g., Bardi et al., 2014). Finally, personal values serve as standards by which we evaluate ourselves and others (Schwartz, 1992) and guide our beliefs, attitudes, and behaviors (e.g., Bardi and Schwartz, 2003; Boer and Fischer, 2013; Sagiv and Roccas, 2021).

**Figure 1:**
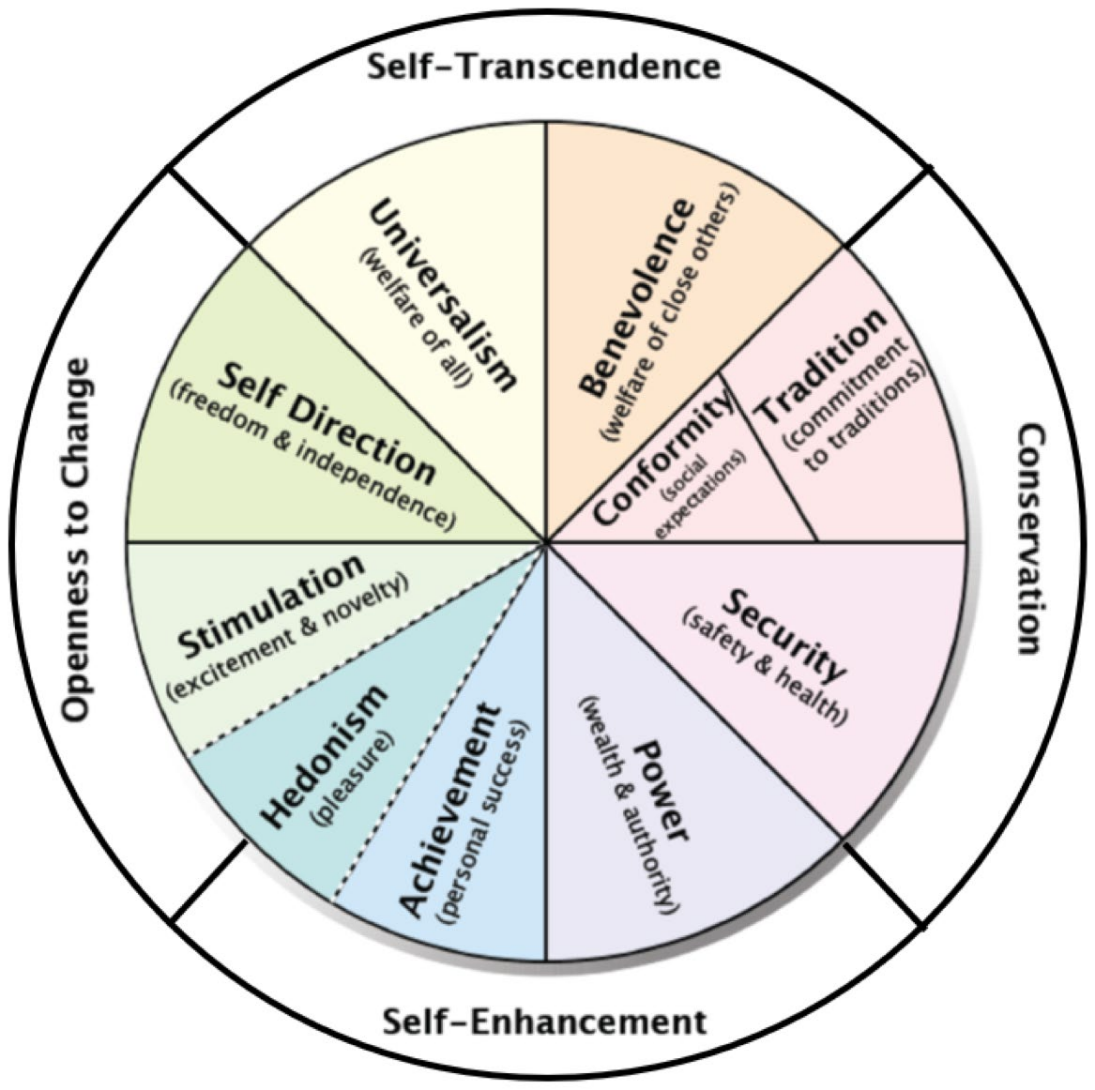
The values circle.

In the present study, we focus on the Schwartz (1992) theory of the content and structure of human values. Schwartz (1992) proposed a theoretical structure of values based on an underlying circular motivational continuum. He partitioned this circular structure into 10 basic values (see Figure 1), with values neighboring each other in the circle (e.g., achievement and power) sharing similar motivations and values opposing each other in the circle having conflicting motivations (e.g., achievement and universalism). Actions that promote one value are likely to promote its neighboring values, but thwart values opposing it in the circle.

Schwartz (1992) summarized the conflicts and compatibilities among the 10 basic values with two bipolar, higher-order value dimensions (see Figure 1). The higher-order value dimension of self-transcendence versus self-enhancement contrasts motivations to prioritize the welfare and interests of others over self-interests (i.e., universalism and benevolence) with the motivation to prioritize self-interests over the interests of others (i.e., achievement, power, and sometimes hedonism). The higher-order value dimension of openness to change versus conservation contrasts the motivation for novelty, change, and autonomy (i.e., self-direction, stimulation, and sometimes hedonism) with the motivation to maintain the status quo (i.e., security, tradition, and conformity).

The circular structure of values shown in Figure 1 has been supported in more than 200 samples in over 80 countries (Sagiv et al., 2017). There is some evidence that people use this circular structure to infer the value priorities of other people that are familiar to them, such as family members or close acquaintances (Skimina and Cieciuch, 2017) and even members of the same society (Hanel et al., 2018). Recent advances in values theory have extended the boundary conditions for the structure of human values to non-human entities (i.e., destinations; Ye et al., 2020). However, little is known about whether people imbue their pets with these more complex and abstract aspects of personality. We propose that:

H1: Pet owners’ perceptions of the values of their pets will reflect the theoretical structure of human values.

### Values similarity and wellbeing

While personal values have been directly associated with subjective wellbeing in adults (e.g., Sagiv and Schwartz, 2000; Bobowik et al., 2011), studies have shown that value congruence, or value similarity, between individuals and their sociocultural environment, can play an important role in wellbeing (e.g., Benish-Weisman et al., 2020; Du et al., 2021). Similarity between peoples’ values and their sociocultural environment has been positively associated with subjective wellbeing (Khaptsova and Schwartz, 2016), relationship satisfaction (Leikas et al., 2018), and self-esteem (Benish-Weisman et al., 2020). Such studies are grounded in theories of person-environment fit, which posit that similarity makes it easier for people to pursue their interests, act on their important values, and express their attitudes and beliefs (Edwards and Rothbard, 1999; Fulmer et al., 2010).

Positive associations between value similarity and subjective wellbeing have been found in a range of relationship contexts, from close others (e.g., romantic partners, Leikas et al., 2018; family members, Hoellger et al., 2021) to distant others (e.g., fellow citizens, Hanel et al., 2020; community members, Sortheix et al., 2013). In addition to the person-environment fit hypothesis described above, similarity with close others may also support the development of closer bonds and more satisfactory relationships (Gaunt, 2006; Hoellger et al., 2021), thus leading to better wellbeing outcomes.

Studies of value similarity and subjective wellbeing have examined similarity at both the value profile and individual values levels. In terms of value profiles, similarity has been positively associated with relationship satisfaction in marital couples (Gaunt, 2006) and with the subjective wellbeing of adult children in parent–child dyads (Hoellger et al., 2021). At the individual values level, conservation values (e.g., Gaunt, 2006; Hanel et al., 2020) and self-enhancement values (e.g., Hanel et al., 2020; Wolf et al., 2021) have generally been positively associated with aspects of wellbeing. However, associations between similarity in openness to change and self-transcendence values and aspects of wellbeing have been more mixed (e.g., Gaunt, 2006; Hanel et al., 2020; Du et al., 2021; Wolf et al., 2021).

While there is some evidence that similarity in owner-pet personality traits is positively associated with owners’ wellbeing (e.g., El-Alayli et al., 2006; Curb et al., 2013), no studies were found to have examined whether people perceive their pets to have humanlike values or whether similarity between owners’ values and their perceptions of their pet’s values is associated with wellbeing outcomes. Given the importance of pets in society, we expect that:

H2: Similarity between pet owners’ values and perceptions of their pet’s values will be positively related to (a) dog owners’ wellbeing and (b) cat owners’ wellbeing.

In the current study, we examine whether pet owners’ perceptions of the value priorities of their pet, in this case a dog or cat, reflect the well-known circular structure of human values shown in Figure 1 (H1). We also examine whether similarity between owners’ personal values and their perceptions of their pet’s values are positively associated with owners’ life satisfaction (H2). In addition to testing our hypotheses, we also explored (a) differences in the value priorities of dog and cat owners, (b) differences in owners’ perceptions of the values of dogs and cats, (c) differences in owner-pet values similarity, and (d) differences in the life satisfaction of dog and cat owners, controlling for age and gender.

## Materials and methods

### Participants and procedures

Two thousand one hundred and sixty-six Australian adult panel members were recruited to complete a survey on animal values. One hundred and eighteen incomplete surveys were removed from the dataset prior to analysis to comply with human ethics approval from the University of Western Australia. Of the remaining 2,048 respondents, 1,279 reported that they owned a pet. Of these respondents, 1,122 reported that they had either a dog or cat as a pet (65% female; *M_age_* = 47 years, *SD* = 15). We excluded the 157 respondents who reported that they had a pet, but not a cat or dog, as totals for other pet types were insufficient for the analysis (see Supplementary material 1). A total of 684 respondents identified themselves as dog owners (61% female; *M_age_* = 46 years, *SD* = 15) and 438 respondents identified themselves as cat owners (71% female; *M_age_* = 47 years, *SD* = 15). The sample sizes for dog and cat owners were sufficient to aim for a small to medium effect size (*f^2^* = 0.02 to *f*^2^ = 0.15; see Supplementary material 2).

The surveys used in the current study were administered to respondents over several weeks, as part of a series of short online surveys. The first survey in the series measured respondent’s personal values. Approximately 2 weeks later, respondents answered questions about their subjective wellbeing and, approximately 3 weeks after that, they answered questions about their pets, including perceptions of their pet’s values.

### Measures

#### Personal values

Respondent’s personal values were measured using the Best-Worst Values Refined scale (BWV-R; Lee et al., 2019). This scale uses best-worst scaling (Louviere et al., 2013), which extends the theory underlying paired comparisons (i.e., Random Utility Theory; Thurstone, 1927) to multiple-choice situations, where each item has a latent value that can be measured by the frequency of choice. Specifically, the BWV-R scale asks respondents to choose the most and the least important values from 21 values sets, derived from a balanced incomplete block experimental design. Based on this design, each value was seen five times, and each pair of values was seen together once (see Lee et al., 2019 for a detailed description of the BWV-R).

The simple count method was used to score respondent’s personal values (see Louviere et al., 2013). This method calculates the score for each value item by subtracting the number of times the item was selected as the least important from the number of times the same item was selected as the most important. This resulted in a −1 to +1 scale, with higher numbers reflecting greater value importance and 0 being the midpoint of the scale.

#### Pet types and values

To identify cat and dog owners, respondents were asked to think about the pet they have had the longest and then select the type of animal (i.e., *Thinking about the pet you have had for the longest, what type of animal is it? cat, dog, fish, bird, small furry animal, farm animal, reptile/amphibian, other*). Respondent’s perceptions of the value priorities of their dog or cat were measured using an adapted version of Lee et al. (2008) values best-worst survey (see Supplementary material 3). In this survey, respondents were asked to think about the pet they have had the longest, as if it were a person. They were then asked to choose the most and least important value for their pet from 11 sets of 6 value items. The 11 sets of value items were derived from a balanced incomplete block experimental design, where each value item appeared 6 times and each pair of items 3 times. The value items were adapted from the Schwartz Values Survey (SVS; Schwartz, 1992), to more directly reflect values that relate to animals. As with the measurement of respondent’s values, the simple count method was used to calculate respondent’s perceptions of their pet’s value priorities, resulting in a −1 to +1 scale. Higher scores reflect greater value importance, with 0 being the midpoint of the scale.

#### Life satisfaction

Respondent’s life satisfaction was measured using Diener et al.’s (1985) Satisfaction with Life Scale (SWLS). Respondents were asked to indicate to what extent they agreed or disagreed with 5 statements (e.g., *in most ways my life is close to my ideal*, *so far I have gotten the important things I want in life*) on a 7-point scale (1 = strongly disagree, 7 = strongly agree). A mean Satisfaction with Life score was obtained for each respondent by averaging their scores for each of the 5 items (α = 0.91).

### Analytical strategy

#### The structure of owners’ and pets’ values

To test H1, multidimensional Scaling (MDS) was used to examine whether the structure of respondent’s personal values and their perceptions of their pet’s values reflect the theorized circular structure of the 10 basic values (see Figure 1). We conducted the MDS using the SPSS version 25 PROXSCAL program with ordinal proximity transformations, Euclidian distance measures, and *z* score transformations of values, in an initial custom configuration of 10 equal points around a circle to estimate the two-dimensional structure (see Schwartz et al., 2012). The initial custom configuration allowed us to compare whether the MDS plots for respondent’s personal values and their perceptions of their pet’s values reflect the theoretical ordering around Schwartz’s (1992) values circle.

#### Preliminary analysis

Prior to undertaking planned analysis, we examined values and life satisfaction for univariate normality using the Kolmogorov–Smirnov test and Quantile-Quantile plots. While the Kolmogorov–Smirnov tests (see Supplementary Table 4B) suggested that owners’ and pets’ values, and life satisfaction were nonnormally distributed, the Q-Q plots showed that data points formed an approximate straight line for each variable (see Supplementary Table 4C). Thus, given the relatively large sample size, we undertook the planned parametric statistical analyses described below.

#### Exploring owner and pet value priorities

Independent samples *t*-tests were used to explore differences in the value priorities of respondents and pets at the sample level. We compared (1) the means of dog owners’ basic values with the means of cat owners’ basic values, and (2) the means of owners’ perceptions of their dog’s basic values with owners’ perceptions of their cat’s basic values.

#### Exploring owner-pet values similarity

To explore whether respondents perceived their pets to have a similar values profile to them, we undertook within-person correlations (see Barni et al., 2011) across all of the 10 basic values. This was done by correlating respondent’s basic values scores with their perceptions of the basic values scores of their pet. Correlations for each respondent were then transformed into *z* scores following Fisher’s *r* to *z* procedure (Kenny and Acitelli, 1994). The *z* scores for each respondent were averaged across the sample to give a mean *z* score and then back transformed to *r* (see Silver and Dunlap, 1987) to assess effect size.

To explore whether respondents perceived their pets to place similar importance on individual basic values as them, we used paired samples *t*-tests. Specifically, we examined differences in mean scores for each of the 10 basic values for dog owners and their dog and cat owners and their cat.

#### Exploring the life satisfaction of pet owners

To explore associations between pet owners’ values and life satisfaction, we used Spearman’s rho rank-order correlations. To examine differences in life satisfaction between dog and cat owners, we used GLM Univariate ANOVA, controlling for respondents age and gender.

#### Owner-pet values similarity and life satisfaction

To examine the similarity between respondent’s own values and their perceptions of the values of their pet, we calculated Owner-Pet Value Incongruence (OPVIC) scores using the generalized absolute difference between respondent’s personal values scores and the values scores of their pet (see Sirgy, 1985; Kressmann et al., 2006; Ye et al., 2020). This method has been suggested to have better predictive validity than other distance models (e.g., Euclidean distance, Sirgy and Samli, 1985; Sirgy, 1985). The mathematical formulation used in the current study follows Sirgy (1985):


$${\mathrm{OPVIC}}_{k}=\frac{\sum_{i=1}^{m}|OV_{ik}-PV_{ik}|}{n}\text{,}$$

where *n* represents the number of value types (*n* = 10), *i* is the specific value type (*i* = self-direction, stimulation, hedonism, etc.), OV*_ik_* is the respondent *k’s* personal value score for the *i* value type (e.g., *k* respondent’s self-direction value), PV*_ik_* is the respondent *k’s* pet values score for the *i* value type (e.g., *k* respondent’s perception of the self-direction values of their pet). OPVIC_k_ is the owner-pet value incongruence score for respondent *k*. A low (vs. high) owner-pet value incongruence score reflects a low (vs. high) averaged absolute value difference score, which, in turn, indicates a high (vs. low) level of similarity between the respondent’s personal values and their perceptions of their pet’s values. To test H2, we ran Pearson’s correlations between OPVIC and mean satisfaction with life scores to examine whether value similarity between owners and their perceptions of their pets’ values was positively associated with life satisfaction.

## Results

### The structure of owner and pet values

**Figure 2:**
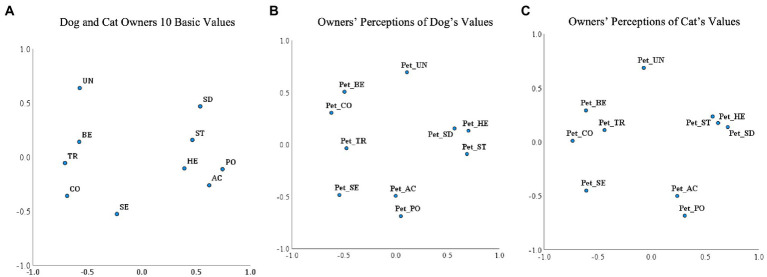
MDS Plots of 10 Basic Values for **(A)** Respondents, **(B)** Dogs, and **(C)** Cats. UN, Universalism; BE, Benevolence; TR, Tradition; CO, Conformity; SE, Security; PO, Power; AC, Achievement; HE, Hedonism; ST, Stimulation; SD, Self-direction.

In support of H1, the MDS shows that people perceive their pets to have values that reflect the theoretical structure of human values (see Figures 2A respondents, Figure 2B dogs, and Figure 2C cats). The three MDS plots fit the data well, with stress measures (Kruskal’s Stress-1) close to zero and DAF (Dispersion Accounted For) and Tucker’s coefficient of congruence close to one (see Figures 2A–C for fit indices). The plots show that for respondents, dogs, and cats the 10 basic values were located in their higher-order value regions (see Figure 1), with only minor deviations in the order of the basic values within these regions.

### Owner and pet value priorities

We explored value priorities for both dog and cat owners and their perceptions of their pet’s values. For both dog and cat owners, benevolence was the most important value (*M* = 0.45, *SD* = 0.30; *M* = 0.45, *SD* = 0.30) and power was the least important value (*M* = −0.52, *SD* = 0.34; *M* = −0.54, *SD* = 0.32) (see Supplementary Table 5). Hedonism was the most important value for both dogs (*M* = 0.41, *SD* = 0.35) and cats (*M* = 0.45, *SD* = 0.33), and power was the least important value for both dogs (*M* = −0.54, *SD* = 0.40) and cats (*M* = −0.42, *SD* = 0.44; see Table 1).

**Table 1:** Means and mean differences in value scores for owners’ perceptions of dogs and cats.

Values	*Dogs*		*Cats*		*t*	*df*	*p*	Cohen’s *d*
	*M*	*SD*	*M*	*SD*				
Self-direction	0.16	0.29	0.32	0.33	−8.30	826.90	<0.001	−0.508
Stimulation	0.13	0.30	0.07	0.30	2.984	1,120	0.003	0.183
Hedonism	0.41	0.35	0.45	0.33	−1.89	977.18	0.059	−0.114
Achievement	−0.17	0.25	−0.14	0.25	−1.97	1,120	0.050	−0.120
Power	−0.54	0.40	−0.42	0.44	−4.51	879.96	<0.001	−0.281
Security	0.23	0.36	0.36	0.40	−5.55	860.51	<0.001	−0.347
Tradition	−0.16	0.30	−0.19	0.28	2.08	1,120	0.037	0.127
Conformity	−0.02	0.41	−0.27	0.43	9.75	897.90	<0.001	0.603
Benevolence	0.25	0.31	0.05	0.28	11.37	1004.06	<0.001	0.679
Universalism	−0.15	0.22	−0.11	0.20	−2.83	1,120	0.005	−0.173

Dogs *n* = 684, Cats *n* = 438. All comparisons were two-tailed, independent samples *t*-tests. df, degrees of freedom.

Independent samples *t*-tests were conducted to compare (1) perceptions of dog and cat values and (2) dog and cat owner’s personal values. There were significant differences in mean scores for all 10 basic values between dogs and cats. Specifically, dogs were perceived by their owners to have higher stimulation, tradition, conformity, and benevolence values than cats, whereas cats were perceived by their owners to have higher self-direction, achievement, power, security, and universalism values than dogs (see Table 1). In contrast, only three differences were found in mean scores for dog and cat owner’s personal values (see Supplementary Table 5), with cat owners reporting higher universalism values (*M* = 0.22 *SD* = 0.24) than dog owners (*M* = 0.18, *SD* = 0.25; *t*(1120) = −2.70, *p* = 0.007) and higher self-direction values (*M* = 0.12 *SD* = 0.27) than dog owners (*M* = 0.08, *SD* = 0.26; *t*(1120) = −2.05, *p* = 0.041). Whereas dog owners reported higher achievement values (*M* = −0.32 *SD* = 0.43) than cat owners (*M* = −0.37, *SD* = 0.43; *t*(1120) = 2.02, *p* = 0.044).

### Exploring owner and pet value similarity

#### Value profile similarity

Across all 10 basic values, within-person correlations between respondent’s values profile and their perceptions of their pet’s values profile varied widely [ranging from *r*_(684) dogs_ = −0.68 to 0.91 and *r*_(438) cats_ = −0.70 to 0.96]. The average within-person correlation between respondent’s values profile and their perceptions of their pet’s values profile was positive and significant (Fisher’s *z*_(684) dogs_ = 0.47, *r*_(684)_ = 0.43, *p* < 0.001; Fisher’s *z*_(438) cats_ = 0.40, *r*_(438)_ = 0.38, *p* < 0.001). The value profile correlations for both dog and cat owners suggest a medium effect size (see Cohen, 1988; Gignac and Szodorai, 2016).

#### Individual value similarity

Positive associations were found between owners’ values and their perceptions of corresponding values for their pet (see Supplementary Table 4A). Specifically, the basic values of dog owners were positively associated with their perceptions of the corresponding value for their dog at the *p* < 0.01 level in 9 out of 10 cases (*r*’s ranged from *r* = 0.11 for stimulation to *r* = 0.35 for power values), the exception being universalism values (*r* = 0.05, *p* = 0.177). For cat owners, 7 out of 10 basic values were positively associated with their perceptions of the corresponding value for their cat at the *p* < 0.01 level (*r*’s ranged from *r* = 0.11 for self-direction to *r* = 0.19 for power values), the exceptions being tradition (*r* = 0.09, *p* = 0.055), benevolence (*r* = 0.05, *p* = 0.350), and universalism (*r* = 0.07, *p* = 0.137) values.

Paired samples *t*-tests found significant differences between owners’ personal values and their perceptions of their pet’s values for most of the 10 basic values. Dog owners perceived their dogs to have significantly higher self-direction, stimulation, hedonism, achievement, tradition, and conformity values and significantly lower security, benevolence, and universalism values than themselves (Table 2, columns 2–7). Cat owners perceived their cats to have significantly higher self-direction, stimulation, hedonism, achievement, power, and tradition values and significantly lower conformity, benevolence, and universalism values than themselves (Table 2, columns 8–15).

**Table 2:** Means and mean differences in values scores for dog owners and dogs and cat owners and cats.

Values	Dog owners		Dogs		*t*	*p*	Cohen’s *d*	Cat owners		Cats		*t*	*p*	Cohen’s *d*
	*M*	*SD*	*M*	*SD*				*M*	*SD*	*M*	*SD*			
Self-direction	0.08	0.26	0.16	0.29	−5.68	<0.001	−0.217	0.12	0.27	0.32	0.34	−10.39	<0.001	−0.496
Stimulation	0.01	0.35	0.13	0.30	−6.99	<0.001	−0.267	−0.02	0.35	0.07	0.30	−4.54	<0.001	−0.217
Hedonism	0.12	0.37	0.41	0.35	−16.28	<0.001	−0.623	0.09	0.36	0.45	0.33	−16.55	<0.001	−0.791
Achievement	−0.32	0.43	−0.17	0.25	−9.35	<0.001	−0.357	−0.37	0.43	−0.14	0.25	−10.83	<0.001	−0.518
Power	−0.52	0.34	−0.54	0.40	1.53	0.126	0.059	−0.54	0.32	−0.42	0.44	−5.02	<0.001	−0.240
Security	0.31	0.26	0.23	0.36	4.85	<0.001	0.185	0.33	0.26	0.36	0.40	−1.71	0.088	−0.082
Tradition	−0.25	0.40	−0.16	0.30	−4.97	<0.001	−0.190	−0.27	0.43	−0.19	0.28	−3.20	<0.001	−0.153
Conformity	−0.13	0.28	−0.02	0.41	−6.52	<0.001	−0.249	−0.17	0.29	−0.27	0.43	4.51	<0.001	0.215
Benevolence	0.45	0.30	0.25	0.31	13.75	<0.001	0.526	0.45	0.30	0.05	0.28	21.34	<0.001	1.020
Universalism	0.18	0.25	−0.15	0.22	26.92	<0.001	1.029	0.22	0.24	−0.11	0.20	23.61	<0.001	1.128

Dog owners and dogs *n* = 684, Cat owners and cats *n* = 438. All comparisons were two-tailed, paired samples *t*-tests. Dog owners and dogs *df* = 683. Cat owners and cats *df* = 437.

### Exploring life satisfaction of pet owners

As relations between owners’ personal values and their life satisfaction were nonlinear, we used Spearman’s rho rank-order correlations to assess relations between these variables for dog and cat owners (see Supplementary Table 6). For dog owners’, life satisfaction was positively associated with their hedonism (*r* = 0.10, *p* = 0.011), tradition (*r* = 0.14, *p* < 0.001), and conformity (*r* = 0.08, *p* = 0.037) values, whereas the basic values of cat owners were not associated with life satisfaction. Owners’ perceptions of their dog’s conformity values were positively associated with their life satisfaction (*r* = 0.10, *p* = 0.010), whereas cat owners’ perceptions of their cat’s basic values were not associated with their life satisfaction.

Results of a GLM Univariate ANOVA (see Supplementary Table 7) indicated that there were significant differences in life satisfaction between dog and cat owners [*F*(1,1,117) = 4.17, *p* = 0.041, η_p_^2^ = 0.004], with dog owners reporting higher life satisfaction than cat owners (*M* = 4.43 and *M* = 4.22, *p* = 0.015, respectively). There were no significant differences in life satisfaction by gender [*F*(1,1,117) = 3.73, *p* = 0.054, η_p_^2^ = 0.003] or by age [*F*(1,1,117) = 0.01, *p* = 0.933, η_p_^2^ = 0.000].

### Owner-pet value similarity and life satisfaction

To test whether the similarity between respondents’ values and their perceptions of their pet’s values was associated with life satisfaction (H2), we compared Owner-Pet Value Incongruence (OPVIC) scores between dog owners and cat owners across all 10 basic values, we then correlated OPVIC scores with life satisfaction for dog and cat owners (see Table 3). We found that OPVIC scores across the basic values were significantly higher for cats (*M* = 0.40, *SD* = 0.10) than for dogs (*M* = 0.36, *SD* = 0.10), *t*(875.203) = −6.17, *p* < 0.001, indicating a higher level of similarity between dog owners’ values and perceptions of their dog’s values, than for cat owners.

**Table 3:** Correlations between OPVIC scores and life satisfaction by pet type and gender.

Values	Dog owners			Cat owners		
	All	Male	Female	All	Male	Female
All basic values	−0.10^**^	−0.11	−0.10^*^	0.05	−0.03	0.08
Self-direction	−0.01	0.02	−0.02	0.08	−0.02	0.11^*^
Stimulation	−0.02	0.02	−0.05	−0.03	0.05	−0.06
Hedonism	−0.16^**^	−0.16^*^	−0.17^**^	0.01	−0.10	0.06
Achievement	−0.08^*^	−0.06	−0.08	−0.03	−0.04	−0.03
Power	−0.06	−0.21^**^	0.01	0.01	−0.04	0.03
Security	0.00	−0.05	0.04	−0.02	0.07	−0.05
Tradition	−0.02	0.03	−0.06	0.03	0.10	−0.02
Conformity	−0.01	0.01	−0.03	0.04	−0.08	0.09
Benevolence	0.03	−0.06	0.10^*^	0.09	−0.06	0.15^**^
Universalism	0.00	0.10	−0.05	−0.02	0.04	−0.05

Dog owners *n* = 684, male dog owners *n* = 266, female dog owners *n* = 418; Cat owners *n* = 438, male cat owners *n* = 126, female cat owners *n* = 312.

* *p* < 0.05.

** *p* < 0.01 (2-tailed).

We found a significant negative association between OPVIC scores and life satisfaction (*r* = −0.10, *p* = 0.008) for dog owners, this suggests that similarity between dog owners’ values and perceptions of their dog’s values had a positive effect on owners’ life satisfaction, supporting H2a. For the 10 basic values, we found negative associations between OPVIC scores and life satisfaction for dog owners for hedonism (*r* = −0.16, *p* < 0.001) and achievement (*r* = −0.08, *p* = 0.049) values, suggesting that similarity in these values was associated with greater life satisfaction for dog owners. However, for cat owners, OPVIC scores were not associated with life satisfaction (*r* = 0.05, *p* = 0.326) (see Table 3), suggesting values similarity had no effect on cat owner’s life satisfaction. Thus, H2b was rejected.

When we examined associations between owner-pet values similarity and life satisfaction by gender, we found significant associations between OPVIC scores for basic values and life satisfaction for female [*r*(416) = −0.10, *p* = 0.05] but not for male [*r*(264) = −0.11, *p* = 0.09] dog owners. This suggests that owner-pet basic values similarity had a positive effect on life satisfaction for female but not male dog owners.

At the individual values level, we found significant negative associations between OPVIC scores and life satisfaction for male dog owners for hedonism [*r*(264) = −0.16, *p* = 0.010] and power [*r*(264) = −0.21, *p* < 0.001] values and for female dog owners for hedonism values [*r*(416) = −0.17, *p* < 0.001]. These findings suggest that greater owner-pet similarity for these values had a positive effect on life satisfaction. Conversely, OPVIC scores for benevolence values were positively associated with life satisfaction in female dog owners [*r*(416) = 0.10, *p* = 0.036], suggesting that for this group, owner-pet similarity in benevolence values had a negative effect on life satisfaction.

For male cat owners, there were no relations between OPVIC scores and life satisfaction for value profiles or individual values. However, while the OPVIC scores for value profiles were not significant for female cat owners, there were significant positive associations for self-direction [*r*(310) = 0.11, *p* = 0.044] and benevolence [*r*(310) = 0.15, *p* = 0.007] values. This suggests that owner-pet similarity in self-direction and benevolence values had a negative effect on life satisfaction for female cat owners.

#### Exploring significant value similarity wellbeing relations

To further examine the significant effects of owner-pet values similarity on life satisfaction, we undertook *an exploratory post hoc* polynomial regression with response surface analysis (see Edwards, 2002). Polynomial regression yields regression coefficients for two linear terms (i.e., pet owner’s personal values and their perceptions of their pet’s values), their multiplicative interaction, and their quadratic terms as predictors of owners’ life satisfaction. The basic model can be specified as follows:

*Life Satisfaction* = *b*_0_ + *b*_1_ *O*+ *b*_2_ *P*+ *b*_3_ *O*^2^+ *b*_4_ *OP* + *b*_5_ *P*^2^ + *e*,

where *O* represents pet owners’ values, *P* represents owners’ perceptions of their pet’s values, *b*_0_ represents the overall intercept, and *e* represents the error term. Studies of values similarity in a range of relationship contexts (e.g., Benish-Weisman et al., 2020; Hanel et al., 2020; Wolf et al., 2021) have demonstrated the utility of polynomial regression and response surface analysis as a useful tool for jointly estimating more complex effects of values similarity on subjective wellbeing.

We ran polynomial regression models for significant associations between owner and pet values similarity (i.e., OPVIC scores) and life satisfaction (see Table 3) for dog and cat owners and for female and male dog and cat owners, separately. First, we ran polynomial regressions for the similarity effects for (1) all dog owners for hedonism and achievement values, (2) male dog owners for hedonism and power values, and (3) female dog owners for hedonism values. In each case, we expected to find a similarity effect based on the negative associations between OPVIC scores and life satisfaction for these values (i.e., the outcome variable will be higher with a greater similarity between predictor variables; Humberg et al., 2019). Second, we ran the same analysis for the *dis*similarity effects for (4) female dog owners for benevolence values and (5) female cat owners for self-direction and benevolence values. In this case, we expected to find a dissimilarity effect based on the positive associations between OPVIC scores and life satisfaction for these values (i.e., the outcome variable will be lower with a greater similarity between predictor variables; Humberg et al., 2019). We then used regression coefficients to construct three-dimensional response surface plots for these relations.

The polynomial regression analysis was undertaken in R (version 3.5.1; R Core Team, 2018), with response surface analysis using the RSA package (Barranti et al., 2017; Schönbrodt et al., 2018; Humberg et al., 2019) and ggplot2 (Wickham et al., 2020). First, we examined response surfaces for congruence (i.e., similarity) or reverse congruence (i.e., dissimilarity) effects between owners’ and pets’ values and life satisfaction.^1^ We then examined regression weights (i.e., *b*_1-5_) separately to obtain more detailed information about similarity and dissimilarity effects (see Hanel et al., 2020).

##### Similarity effects

**Figure 3:**
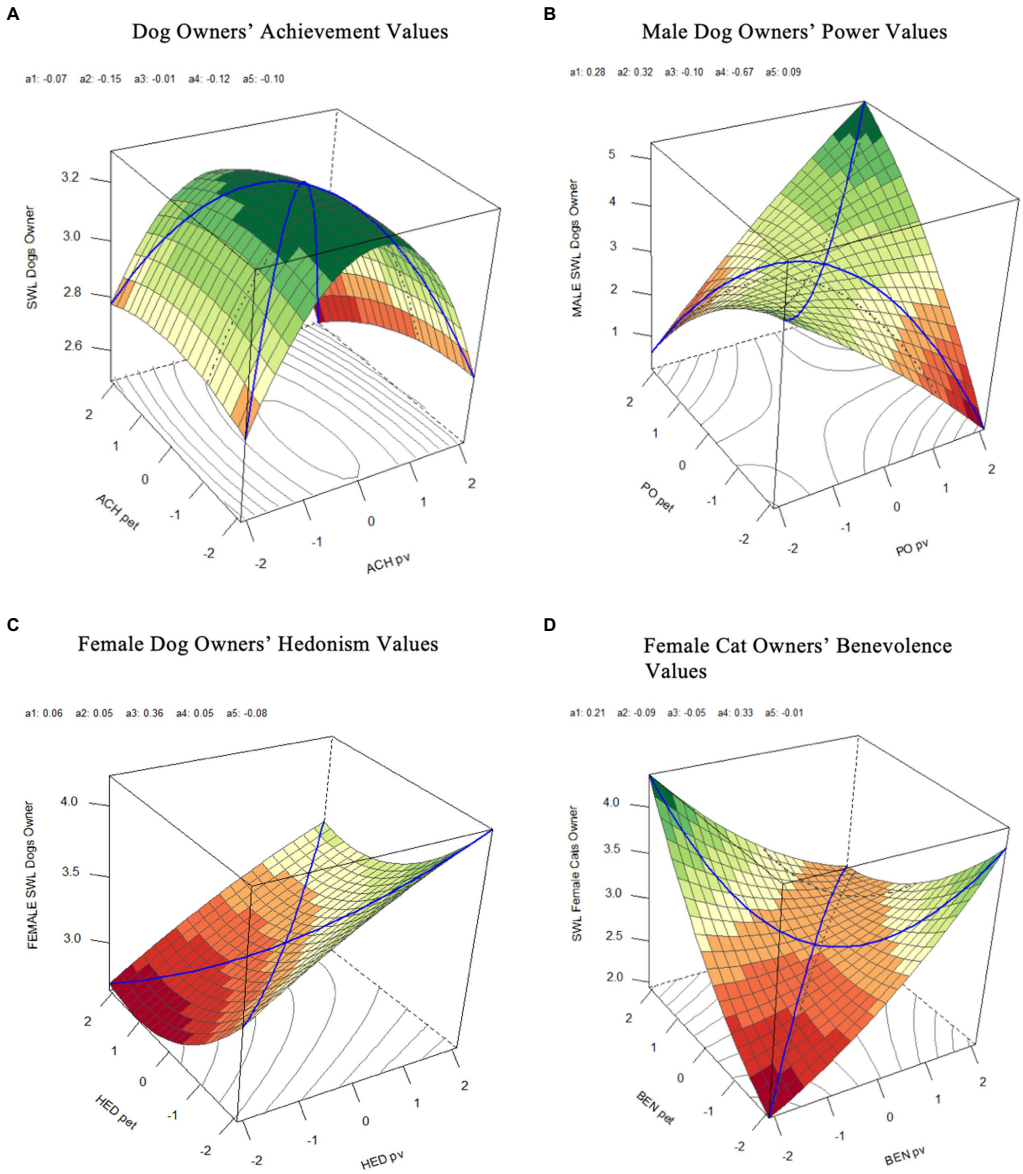
Response surface plots for **(A)** dog owner’s achievement values, **(B)** male dog owners power values, **(C)** female dog owner’s hedonism values, and **(D)** female cat owner’s benevolence values. SWL, satisfaction with life; pv, owners’ values; pet, pet’s values; ACH, achievement values; PO, power values; HED, hedonism values; BEN, benevolence values.

Neither hedonism nor achievement values for all dog owners satisfied Humberg et al. (2019) conditions for a congruence effect (see Supplementary Table 8). However, for achievement values, we found a significant, negative quadratic effect of owner’s values on life satisfaction (*b* = −0.56, *SE* = 0.28, *p* = 0.048). This reflects the inverted U-shape of the line of congruence shown in the surface plot (Figure 3A), suggesting that higher levels of life satisfaction occurred when owners’ and dogs’ achievement values were similar at the midrange of value importance, rather than at low or high levels of importance.

The response surface for male dog owners’ power values met the conditions for a similarity effect (see Figure 3B; Supplementary Table 8), with a significantly negative surface parameter *a*_4_ = −3.02, *p* = 0.013. This suggests that male dog owners will have greater life satisfaction the more similar their power values are with their perceptions of the power values of their dog. The positive, significant interaction term (*b* = 2.31, *SE* = 0.63, *p* < 0.001) also suggests that higher levels of life satisfaction occurred when owners higher in power values perceived their dogs to be higher in power values.

As with hedonism values for all dog owners, the response surface for male and female dog owners did not meet the conditions for a similarity effect (see Supplementary Table 8). However, for female dog owners, there was a personal value effect (*b* = 0.85, *SE* = 0.35, *p* = 0.014), suggesting that higher levels of life satisfaction occurred as owners’ hedonism values increased, regardless of their perceptions of their dog’s hedonism values (see Figure 3C).

##### Dissimilarity effects

The response surface for female cat owner’s benevolence values met the conditions for a dissimilarity effect (see Figure 3D; Supplementary Table 8), with a significant positive surface parameter *a*_4_ = 2.91, *p* = 0.026. This suggests that female cat owners will have lower life satisfaction the more similar their benevolence values are with their perceptions of their cat’s benevolence values. As can be seen in the surface plot, the highest levels of life satisfaction occurred when owner’s benevolence values were high and cats were low, and when cat’s benevolence values were high and owners were low. This pattern is supported by the significant, negative interaction between owners and cats’ values (*b* = −1.96, *SE* = 0.97, *p* = 0.043). The response surface for female dog owners for benevolence values and female cat owners for self-direction values did not meet the conditions for a dissimilarity effect (see Supplementary Table 8).

## Discussion

We found that owners imbued their pets with humanlike values in a way that reflects the complex circular structure of Schwartz’s (1992) basic values. This finding extends previous studies of anthropomorphic inferences about the personality of non-human animals beyond lists of traits (e.g., Cavanaugh et al., 2008; Epley et al., 2008; Litchfield et al., 2017; McConnell et al., 2019) to a more complex set of conflicts and compatibilities that underlie the structure of the 10 basic values.

While there was some evidence that owners project their own value profiles on to their pets, mean differences in 9 out of 10 basic values for dog owners and dogs and cat owners and cats, suggests that owners’ perceptions of their pet’s values are not only grounded in self-projection. It may be the case that owners’ perceptions of their pet’s values are based on a combination of their own mental states and characteristics (e.g., Turcsán et al., 2012), their perceptions of other humans (e.g., Kwan et al., 2008), observations of actual pet behaviors that may express those characteristics (e.g., Gosling et al., 2003), and stereotypes about the animal or breed (e.g., Kwan et al., 2008). Further research is required to untangle these possible effects on perceptions of pet’s values. Future studies could examine whether pet owners perceive popular breeds to have particular value profiles, and whether length of ownership and closeness with a pet influences owners’ perception of their pet’s values.

While there were very few differences between the values of dog and cat owners, their perceptions of the values of their dog or cat differed for 9 out of the 10 basic values in a systematic pattern. Specifically, dogs were perceived to have higher social-focus values that emphasize concern with outcomes for others (i.e., tradition, conformity, and benevolence) than cats, whereas cats were perceived to have higher personal-focus values that emphasize concern with outcomes for oneself (i.e., self-direction, achievement, and power) than dogs. These differences in patterns of perceived value priorities for dogs and cats have elements in common with other studies of pet personality. For example, dogs were perceived to be more sociable and protective than cats (Serpell, 1996; Menchetti et al., 2018), whereas cats were perceived to be more independent and neurotic than dogs (Miklósi et al., 2005; Menchetti et al., 2018). Differences in people’s perceptions of the personalities of dogs and cats may reflect more widely held beliefs about species-specific characteristics that are grounded in differences in the domestication histories of dogs and cats (Arahori et al., 2017; Menchetti et al., 2018). To explore this further, future studies could examine whether both pet owners and people without pets perceive dogs and cats to have distinct value profiles.

Dog owners reported higher life satisfaction than cat owners. These differences reflect Bao and Schreer’s (2016) finding that dog owners reported higher wellbeing than cat owners (see also Wilkins et al., 2020). They found that higher wellbeing in dog owners could be partly explained by differences in owner personality traits. Specifically, they found that dog owners had higher levels of agreeableness and extraversion than cat owners, and that these traits positively predicted aspects of wellbeing. We extend these findings to values. Specifically, we found that tradition and conformity values (associated with agreeableness, Parks-Leduc et al., 2015) and hedonism values (associated with extraversion, Parks-Leduc et al., 2015), were positively associated with life satisfaction for dog owners but not for cat owners.

In addition to personality, it may also be the case that differences in the relationship that owners have with their pet dog or cat may influence wellbeing outcomes. Dog owners have been found to be more likely to consider their pets as family members than cat owners (Arahori et al., 2017), and dogs are perceived by their owners to have a more positive impact on promoting morale and interaction in the family than cats (Albert and Anderson, 1997). Previous studies have also found that owners reported greater emotional closeness with dogs than cats (González-Ramírez and Landero-Hernández, 2021) and that dog ownership appeared to act as a buffer against loneliness, but cat ownership did not (Oliva and Johnston, 2021). Future research should consider whether relationship quality moderates or mediates relations between values and wellbeing in pet owners.

Across the 10 basic values, we found a higher level of similarity between dog owners’ values and perceptions of their dog’s values, than for cat owners and their cats. We also found that values similarity was associated with life satisfaction for dog owners’ but not for cat owners. This may be related to the different relationships that people have with their pet dog or cat. Dog owners tend to have closer psychological distance (Arahori et al., 2017) and emotional closeness (González-Ramírez and Landero-Hernández, 2021) with their dogs than cat owners do with their cats.

At the individual value level, similarity in hedonism and achievement values was positively associated with life satisfaction for dog owners. Similarity in these values between humans has previously been found to relate to wellbeing (e.g., similarity with others in society, Hanel et al., 2020). Further, Leikas et al. (2018) found similarity with a spouse in hedonism values was positively associated with relationship satisfaction. These personal-focus values emphasize outcomes for the self rather than for others and have mixed direct associations with subjective wellbeing (Schwartz and Sortheix, 2018). However, it is posited that direct relations between values and wellbeing have different mechanisms to those associated with person-environment fit (Sagiv and Schwartz, 2000). Therefore, we used polynomial regression analysis with response surface plots to jointly examine both direct and indirect (i.e., person-environment fit) associations between values and life satisfaction.

The results of the polynomial regression and response surface analysis showed similarity effects at high and low levels of power value importance for male dog owners. This indicated that (a) male dog owners who place a high importance on power values are more satisfied with their life when they perceive their dog to place an equally high importance on power values and (b) male dog owners that place a low importance on power values may be more satisfied with their life when they perceive their dog to also place a low importance on power values. People who prioritize power values tend to be more dominant and competitive (e.g., Cohrs et al., 2005; Sagiv et al., 2011) than those who do not. In this context, matching power values at high vs. low levels of importance may manifest in owner’s selection preferences for more versus less powerful breeds of dog.

Dissimilarity effects at high and low levels of benevolence values in the female cat owner group may reflect the content of these values. It may be the case that female cat owners high on benevolence values have higher life satisfaction when they perceive that their cat does not prioritize these values, as this may provide them with the opportunity to attain valued goals through nurturing and caring for their cat. Conversely, female cat owners who place a relatively low importance on benevolence values may be more satisfied with their life if their cat prioritizes these values, as they may feel that their cat is nurturing and caring for them.

Some potential limitations should also be acknowledged. Self-reports were used to measure personal values and owners’ perceptions of their pet’s values. While self-reports are commonly used in personal values research (e.g., Lee et al., 2022), it may be the case that this approach amplified owner-pet values similarity. Future studies could consider including peer ratings of owner and pet values, in a similar way to studies examining pet personality traits (e.g., Gosling et al., 2003). Caution should also be taken with the generalization of these results, as much of our analysis was necessarily exploratory in nature, as the first study into owners’ perceptions of their pet’s values. More research is needed to replicate and extend our results to other samples and pet types. Further, while we examined the effects of age and gender on owner’s wellbeing, there may be other factors that influence wellbeing and values-wellbeing relations (e.g., income, employment status, relationship status, social support, and family structure). Future studies should control for these factors and examine potential moderating effects of pet characteristics (e.g., the number, age, breed, health of pets) on relations between values and wellbeing.

## Conclusion

People imbue their pet dogs and cats with humanlike values and these perceptions are not simply the projection of owners’ personal values onto their dog or cat. Patterns of differences in the values attributed to dogs and cats suggest that people perceive dogs to prioritize more social-focus values than cats and cats to prioritize more personal-focus values than dogs. Similarity between owners’ personal value profiles and their perceptions of their dog’s values profile had a positive impact on owners’ life satisfaction, but this was not the case for cat owners’ and their cats. However, associations between individual values similarity and life satisfaction were much more nuanced when we jointly examined direct and indirect effects.

The current study contributes to our understanding of the direct and indirect effects of personal values on subjective wellbeing, as well as the role of pets in the wellbeing outcomes of their owners. It also enriches our understanding of the anthropomorphic inferences that people make about their pets by extending the attribution of animal personality beyond lists of traits to the more complex structure of human values. As such, the current research should help to lay the foundation for future studies that seek to understand how peoples’ perceptions of animals can contribute to human psychology.

## Data availability statement

The raw data supporting the conclusions of this article will be made available by the authors, without undue reservation.

## Ethics statement

The studies involving human participants were reviewed and approved by the University of Western Australia Human Ethics Committee and complies with the National Statement on Ethical Conduct in Human Research (2007, updated 2018). The patients/participants provided their written informed consent to participate in this study.

## Author contributions

All authors listed have made a substantial, direct, and intellectual contribution to the work and approved it for publication.

## Funding

This research was funded by an Australian Research Council Linkage grant in partnership with Pureprofile (LP150100434) and supported by grants from the National Natural Science Foundation of China (72102070), and the Shanghai Planning Office of Philosophy and Social Science (2021BGL003).

## Conflict of interest

The authors declare that this study received funding from Pureprofile in partnership with an Australian Research Council Linkage Grant. The funder had the following involvement in the study: data collection was conducted in the Pureprofile panel.

## Publisher’s note

All claims expressed in this article are solely those of the authors and do not necessarily represent those of their affiliated organizations, or those of the publisher, the editors and the reviewers. Any product that may be evaluated in this article, or claim that may be made by its manufacturer, is not guaranteed or endorsed by the publisher.

## Footnotes

^1^As outlined in Humberg et al. (2019, p.415, Figure 3) a response surface should meet the following conditions for a congruence effect: (1) The first principle axis should not differ from the line of congruence (i.e., *p*_10_ ≈ 0 and *p*_11_ ≈ 1), and (2) the surface above the line of incongruence should be an inverted U-shape and have a nonsignificant slope above the origin (i.e., *a*_4_ < 0 and *a*_3_ ≈ 0). For a *reverse* congruence effect a response surface should meet the following conditions: (3) The second principle axis should not differ from the line of congruence (i.e., *p*_20_ ≈ 0 and *p*_21_ ≈ 1), and (4) *a*_4_ > 0 and *a*_3_ ≈ 0).
