# Supplementary material for: The effect of similarity between owner’s values and their perceptions of their pet’s values on life satisfaction
Source: Front Psychol. 2022 Oct 28;13:1029883. doi: 10.3389/fpsyg.2022.1029883 (PMC9651006; doi:10.3389/fpsyg.2022.1029883)
Supplement: Supplementary file 1 [file Data_Sheet_1.docx]

Supplementary Material

# Pet Types

| Pet Type | *N* | % |
| --- | --- | --- |
| Dog | 684 | 53.5 |
| Cat | 438 | 34.2 |
| Fish | 43 | 3.4 |
| Bird | 55 | 4.3 |
| Small furry animals (e.g., hamster, ferret, gerbil, guinea pig, mouse, rat, rabbit) | 28 | 2.2 |
| Farm animals (e.g., horse, goat, pig) | 11 | 0.9 |
| Reptile or amphibian (e.g., tortoise, snake, lizard, frog, newt) | 11 | 0.9 |
| Other | 9 | 0.7 |
| Total | 1,279 | 100 |

# Power Analysis

Using the G*Power program (Buchner et al., 2013) for an F test to detect a change in R^2^, a priori power analysis suggested a sample size of 92 would be a statistical power 1-β = 0.80 at α = .05, for a medium effect size *f^2^*= 0.15. A post hoc analysis suggested a statistical power ranging from 1-β = 1.00 to 1-β = 0.99 at α = .05 for the dog (*n* = 684) and cat owner samples (*n* = 438). The sensitivity analysis showed that the sample size (*n*_dog_ = 684, *n*_cat_ = 438) was large enough to generate small effect sizes *f^2^*_dog_ = 0.02 and *f^2^*_cat_ = 0.03 at the power = 80% and α = .05.

**References**

Buchner, A., Erdfelder, E., Faul, F., & Lang, A. G. (2013). G* Power 3: Statistical power analysis program for sample size estimation (3.1.7)[Computer program and user guide].

# Pet Values

Pet values were measured with a best-worst scale adapted from Lee et al. (2008). This scale asks participants to choose the MOST and the LEAST important basic values for the pet from 11 value sets derived from a balanced incomplete block design. The full scale is provided below.

1/11) Of these, which are the MOST and the LEAST important to your PET, as guiding principles in life?

| Most Important |  | Least Important |
| --- | --- | --- |
|  | Safety and Stability |  |
|  | Humble and respecting traditions |  |
|  | Helpful and loyal |  |
|  | Fairness for all |  |
|  | Following rules and being obedient |  |
|  | Unity with nature |  |

2/11) Of these, which are the MOST and the LEAST important to your PET, as guiding principles in life?

| Most Important |  | Least Important |
| --- | --- | --- |
|  | An exciting and varied life |  |
|  | Freedom and curiosity |  |
|  | Helpful and loyal |  |
|  | Fairness for all |  |
|  | Social status and prestige |  |
|  | Unity with nature |  |

3/11) Of these, which are the MOST and the LEAST important to your PET, as guiding principles in life?

| Most Important |  | Least Important |
| --- | --- | --- |
|  | An exciting and varied life |  |
|  | Safety and stability |  |
|  | Pleasure and enjoying life  Fairness for all |  |
|  | Social status and prestige |  |
|  | Following rules and being obedient |  |

4/11) Of these, which are the MOST and the LEAST important to your PET, as guiding principles in life?

| Most Important |  | Least Important |
| --- | --- | --- |
|  | Safety and Stability |  |
|  | Freedom and curiosity |  |
|  | Capable and ambitious |  |
|  | Social status and prestige |  |
|  | Following rules and being obedient |  |
|  | Unity with nature |  |

5/11) Of these, which are the MOST and the LEAST important to your PET, as guiding principles in life?

| Most Important |  | Least Important |
| --- | --- | --- |
|  | An exciting and varied life |  |
|  | Freedom and curiosity |  |
|  | Pleasure and enjoying life |  |
|  | Humble and respecting traditions |  |
|  | Following rules and being obedient |  |
|  | Unity with nature |  |

6/11) Of these, which are the MOST and the LEAST important to your PET, as guiding principles in life?

| Most Important |  | Least Important |
| --- | --- | --- |
|  | An exciting and varied life |  |
|  | Safety and stability |  |
|  | Pleasure and enjoying life |  |
|  | Capable and ambitious |  |
|  | Helpful and loyal  Unity with nature |  |

7/11) Of these, which are the MOST and the LEAST important to your PET, as guiding principles in life?

| Most Important |  | Least Important |
| --- | --- | --- |
|  | An exciting and varied life |  |
|  | Safety and stability |  |
|  | Freedom and curiosity |  |
|  | Capable and ambitious |  |
|  | Humble and respecting traditions |  |
|  | Fairness for all |  |

8/11) Of these, which are the MOST and the LEAST important to your PET, as guiding principles in life?

| Most Important |  | Least Important |
| --- | --- | --- |
|  | Safety and stability |  |
|  | Freedom and curiosity |  |
|  | Pleasure and enjoying life |  |
|  | Humble and respecting traditions |  |
|  | Helpful and loyal |  |
|  | Social status and prestige |  |

9/11) Of these, which are the MOST and the LEAST important to your PET, as guiding principles in life?

| Most Important |  | Least Important |
| --- | --- | --- |
|  | Freedom and curiosity |  |
|  | Pleasure and enjoying life |  |
|  | Capable and ambitious |  |
|  | Helpful and loyal |  |
|  | Fairness for all |  |
|  | Following rules and being obedient |  |

10/11) Of these, which are the MOST and the LEAST important to your PET, as guiding principles in life?

| Most Important |  | Least Important |
| --- | --- | --- |
|  | Pleasure and enjoying life |  |
|  | Capable and ambitious |  |
|  | Humble and respecting traditions |  |
|  | Fairness for all |  |
|  | Social status and prestige |  |
|  | Unity with nature |  |

11/11) Of these, which are the MOST and the LEAST important to your PET, as guiding principles in life?

| Most Important |  | Least Important |
| --- | --- | --- |
|  | An exciting and varied life |  |
|  | Capable and ambitious |  |
|  | Humble and respecting traditions |  |
|  | Helpful and loyal |  |
|  | Social status and prestige |  |
|  | Following rules and being obedient |  |

# Preliminary Analysis

**Supplementary Table 4a.** Intercorrelations Between Study Variables.

|  |  | Owner values | | | | | | | | | | |
| --- | --- | --- | --- | --- | --- | --- | --- | --- | --- | --- | --- | --- |
|  |  | 1. | | 2. | 3. | 4. | 5. | 6. | 7. | 8. | 9. | 10. |
| 1. Self-direction | Dogs | **.12**^**^ | .04 | | -.02 | -.05 | -.16**^**^** | .01 | -.05 | -.09**^*^** | .17**^**^** | .10**^**^** |
|  | Cats | **.11**^*^ | .08 | | .02 | -.17^**^ | -.20^**^ | .01 | .05 | -.11^**^ | .10^*^ | .13^*^ |
| 2. Stimulation | Dogs | .07 | **.11**^**^ | | .06 | .02 | -.04 | .04 | -.07 | -.06 | .02 | .04 |
|  | Cats | .06 | **.14**^**^ | | .12^*^ | .06 | .03 | .02 | -.06 | -.09 | .04 | -.07 |
| 3. Hedonism | Dogs | .08^*^ | .08^*^ | | **.13**^**^ | -.14^**^ | -.29^**^ | .19^**^ | -.13^**^ | -.12^**^ | .31^**^ | .10^**^ |
|  | Cats | .12^*^ | .08 | | **.12**^**^ | -.09 | -.10^*^ | .03 | -.13^**^ | -.06 | .05 | .06 |
| 4. Achievement | Dogs | .05 | .09^*^ | | .04 | **.30**^**^ | .24^**^ | -.21^**^ | .04 | -.10^*^ | -.24^**^ | -.15^**^ |
|  | Cats | -.01 | .02 | | -.00 | **.15**^**^ | .10^*^ | -.10^*^ | -.06 | .01 | -.05 | -.06 |
| 5. Power | Dogs | -.06 | .02 | | .06 | .24^**^ | **.35**^**^ | -.21^**^ | -.00 | .02 | -.35^**^ | -.15^**^ |
|  | Cats | .06 | .02 | | -.04 | .06 | **.19**^**^ | -.04 | -.07 | -.02 | -.15^**^ | -.01 |
| 6. Security | Dogs | -.02 | | -.08^*^ | -.05 | -.13^**^ | -.15^**^ | **.18**^**^ | -.01 | -.02 | .14^**^ | .08^**^ |
|  | Cats | .03 | | -.10^*^ | -.08 | -.07 | -.15^**^ | **.14**^**^ | .01 | .00 | .03 | .07 |
| 7. Tradition | Dogs | -.07 | | -.06 | -.06 | .06 | .10^*^ | -.09^*^ | **.12**^**^ | .09^*^ | -.12^**^ | -.08^**^ |
|  | Cats | -.14^**^ | | -.01 | -.03 | .01 | .07 | -.01 | **.09** | .01 | -.02 | -.07 |
| 8. Conformity | Dogs | -.08^*^ | | .01 | -.03 | -.04 | -.03 | .07 | -.00 | **.13**^**^ | .03 | -.05 |
|  | Cats | -.15^**^ | | -.09 | -.01 | .09^*^ | .11^*^ | .01 | .07 | **.14**^**^ | .02 | -.15^**^ |
| 9. Benevolence | Dogs | .01 | | -.09^*^ | -.04 | -.11^**^ | -.12^**^ | .07 | -.01 | .01 | **.19**^**^ | .04 |
|  | Cats | -.02 | | -.05 | -.02 | .06 | -.05 | -.05 | -.04 | .05 | **.05** | .02 |
| 10. Universalism | Dogs | -.04 | | -.07 | -.08^*^ | -.09^*^ | .07 | -.08 | -.09^*^ | .06 | -.12^**^ | **.05** |
|  | Cats | -.06 | | -.03 | -.03 | -.08 | -.03 | -.05 | .10^*^ | .05 | -.03 | **.07** |

*Note.* Dog Owners and Dogs *n* = 684, Cat Owners and Cats *n* = 438; ^*^ *p* < .01, ^**^ *p* < .001.

## Assumption Checks for Planned Analysis

**Supplementary Table 4b.** Kolmogorov-Smirnov Test of Normality for Owners and Pets Values and Life Satisfaction.

|  |  | Pet Owners Values | | | Pets Values | | |
| --- | --- | --- | --- | --- | --- | --- | --- |
|  |  | Statistic | df | *p* | Statistic | df | *p* |
| Self-direction | Dog | 0.122 | 684 | <.001 | 0.173 | 684 | <.001 |
|  | Cat | 0.122 | 438 | <.001 | 0.126 | 438 | <.001 |
| Stimulation | Dog | 0.152 | 684 | <.001 | 0.152 | 684 | <.001 |
|  | Cat | 0.151 | 438 | <.001 | 0.182 | 438 | <.001 |
| Hedonism | Dog | 0.113 | 684 | <.001 | 0.095 | 684 | <.001 |
|  | Cat | 0.134 | 438 | <.001 | 0.122 | 438 | <.001 |
| Achievement | Dog | 0.132 | 684 | <.001 | 0.141 | 684 | <.001 |
|  | Cat | 0.173 | 438 | <.001 | 0.178 | 438 | <.001 |
| Power | Dog | 0.167 | 684 | <.001 | 0.162 | 684 | <.001 |
|  | Cat | 0.155 | 438 | <.001 | 0.119 | 438 | <.001 |
| Security | Dog | 0.096 | 684 | <.001 | 0.165 | 684 | <.001 |
|  | Cat | 0.100 | 438 | <.001 | 0.160 | 438 | <.001 |
| Tradition | Dog | 0.141 | 684 | <.001 | 0.142 | 684 | <.001 |
|  | Cat | 0.110 | 438 | <.001 | 0.138 | 438 | <.001 |
| Conformity | Dog | 0.114 | 684 | <.001 | 0.179 | 684 | <.001 |
|  | Cat | 0.098 | 438 | <.001 | 0.126 | 438 | <.001 |
| Benevolence | Dog | 0.103 | 684 | <.001 | 0.191 | 684 | <.001 |
|  | Cat | 0.099 | 438 | <.001 | 0.193 | 438 | <.001 |
| Universalism | Dog | 0.081 | 684 | <.001 | 0.122 | 684 | <.001 |
|  | Cat | 0.071 | 438 | <.001 | 0.140 | 438 | <.001 |
| Life Satisfaction | .083 | 1122 | <.001 |  |  |  |  |

*Note*. Kolmogorov-Smirnov test with Lilliefors Correction. Significant results suggest deviation from normality.

| **Supplementary Figure 4c.** Quantile-Quantile Plots of Owners and Pets Values. | | | |
| --- | --- | --- | --- |
| Dog Owners | Dogs | Cat Owners | Cats |
| Self-direction | | | |
| 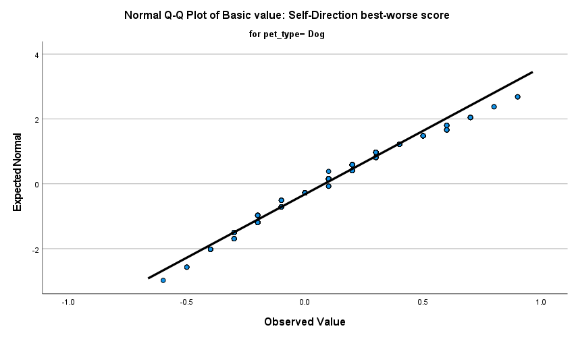 | 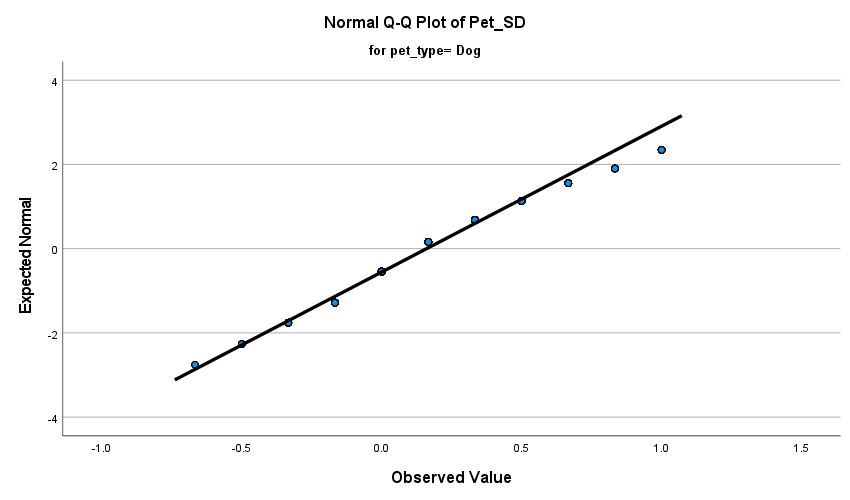 | 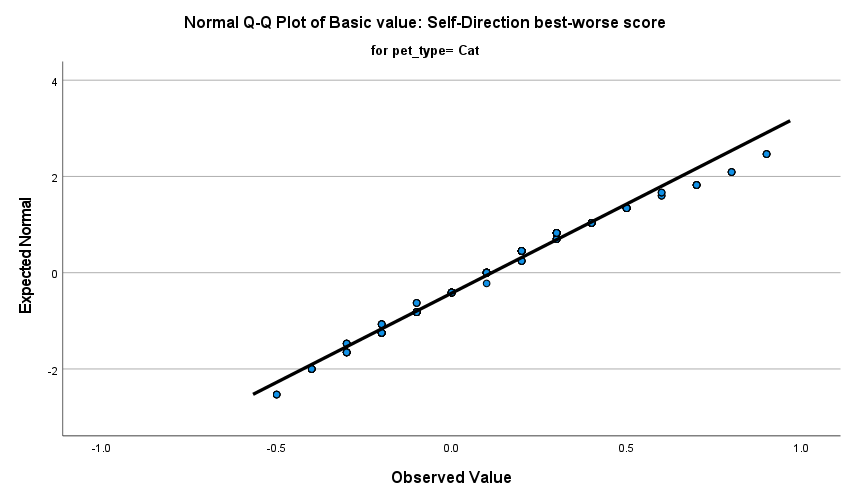 | 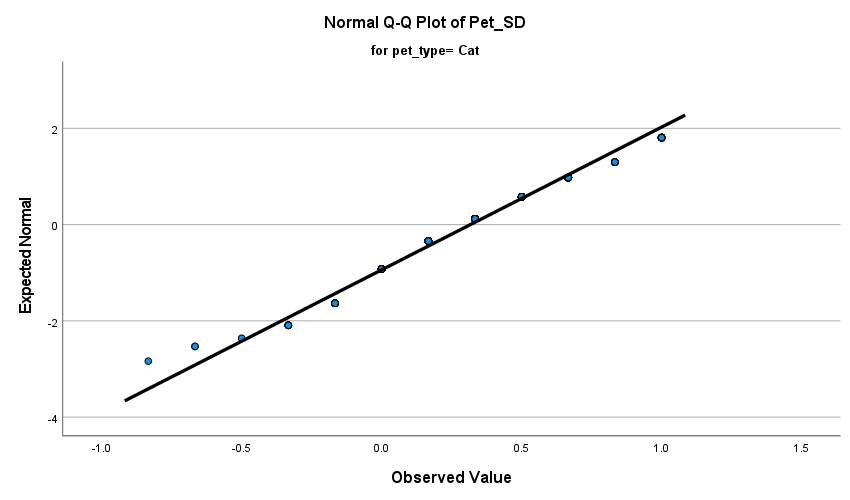 |
| Stimulation | | | |
| 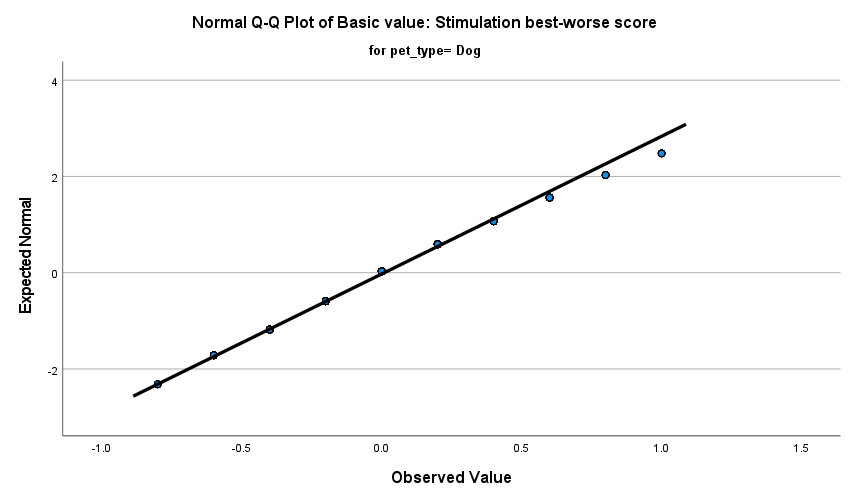 | 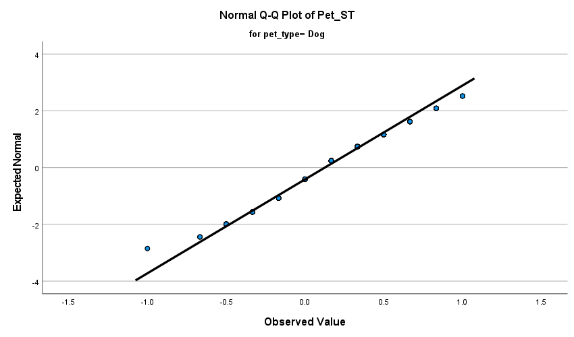 | 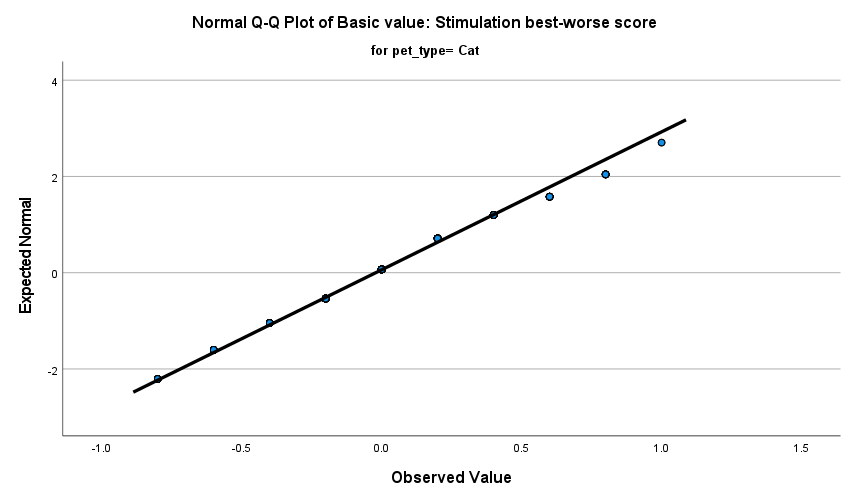 | 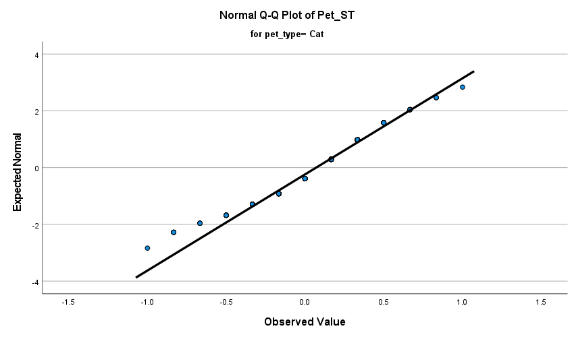 |
| Hedonism | | | |
| 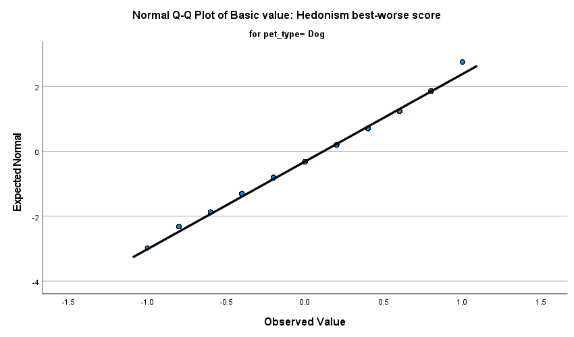 | 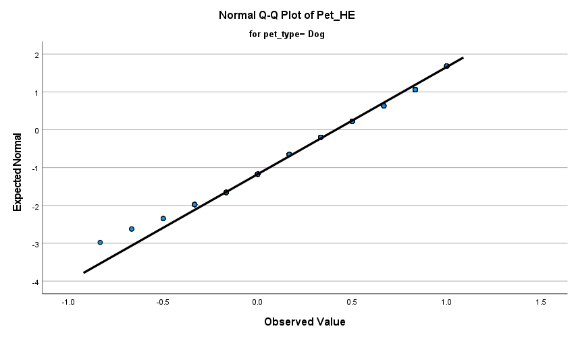 | 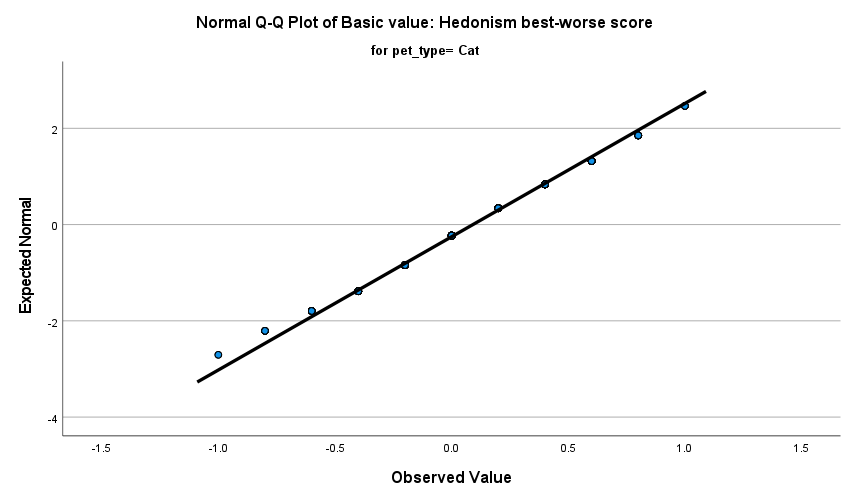 | 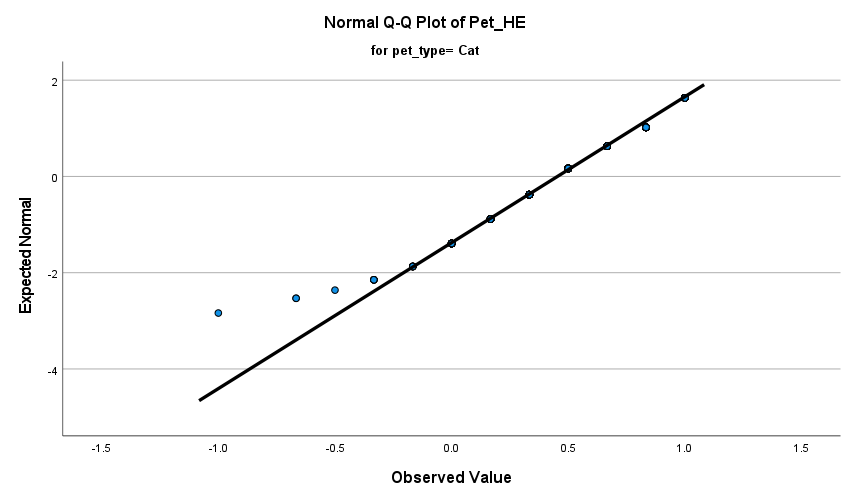 |
| Achievement | | | |
| 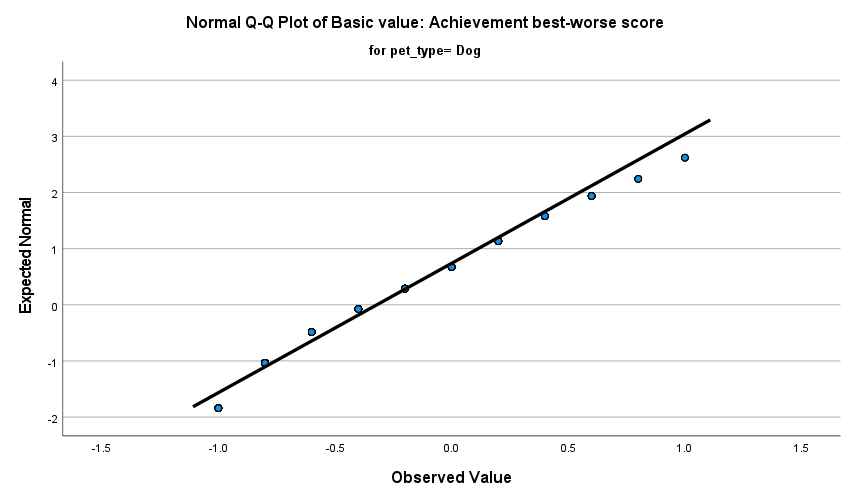 | 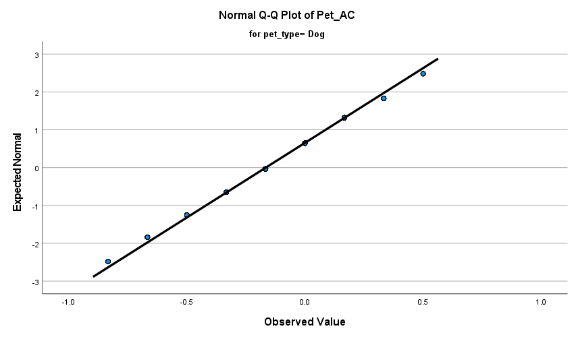 | 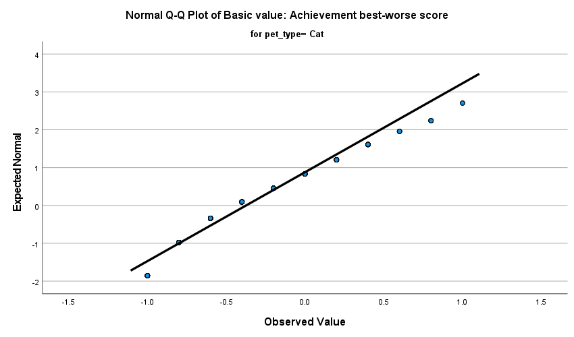 | 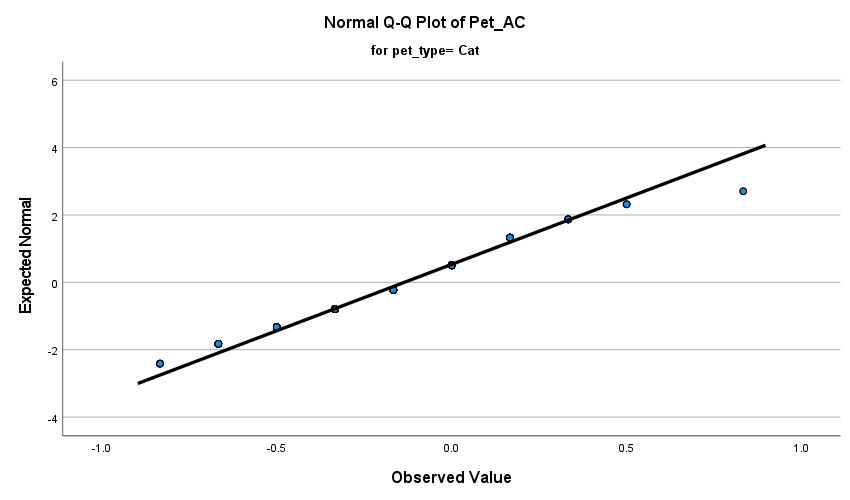 |
| Power | | | |
| 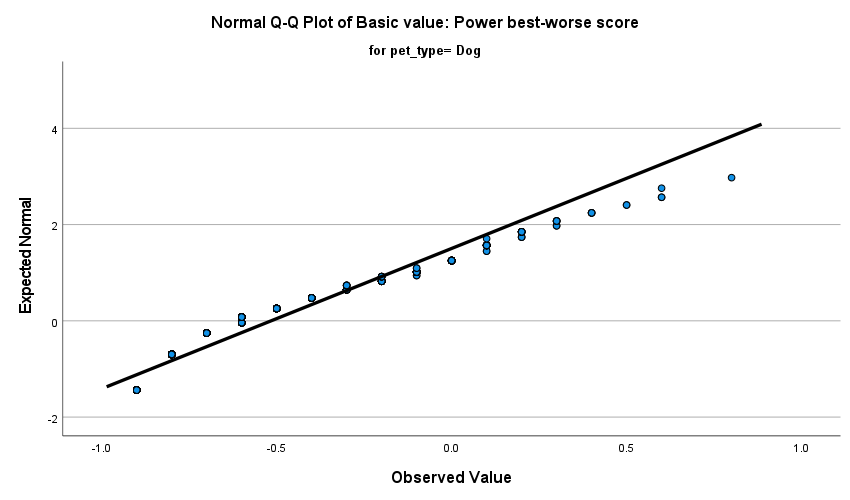 | 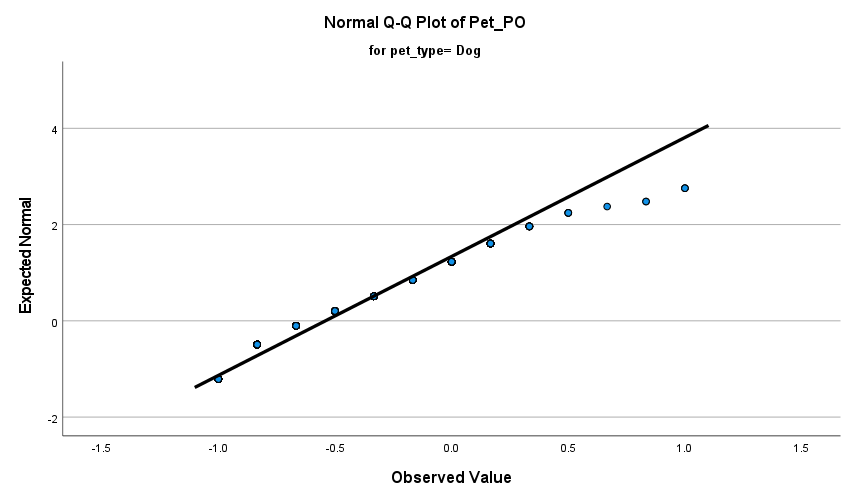 | 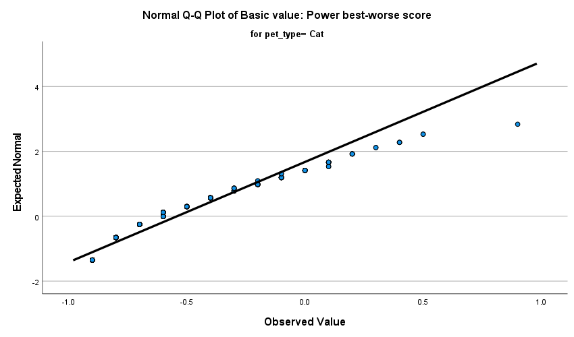 | 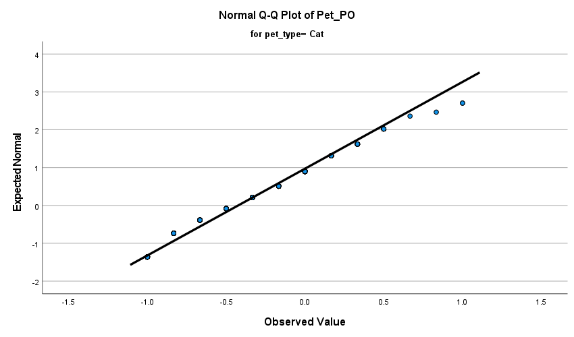 |
| Security | | | |
| 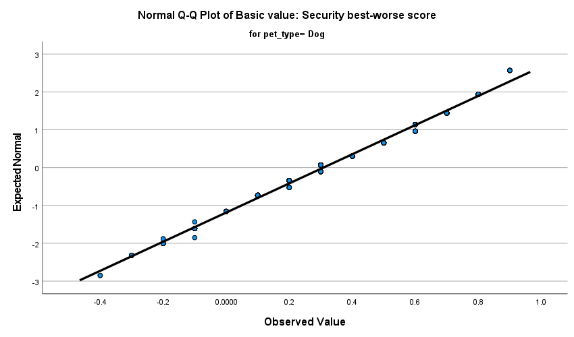 | 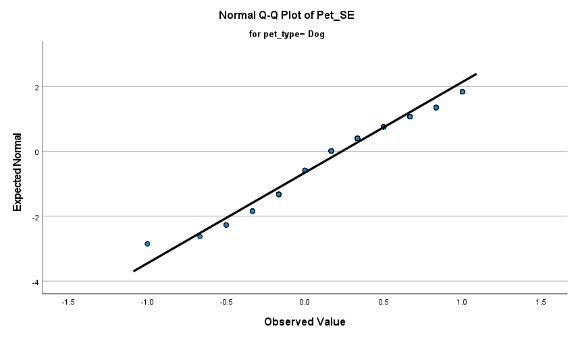 | 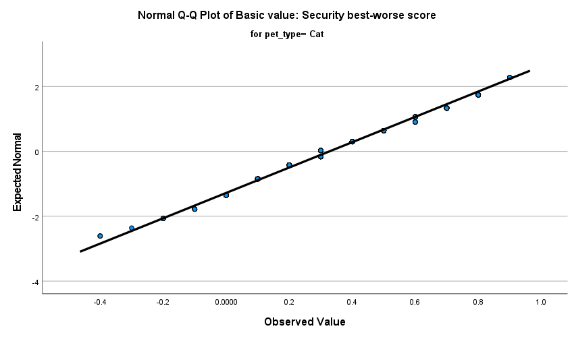 | 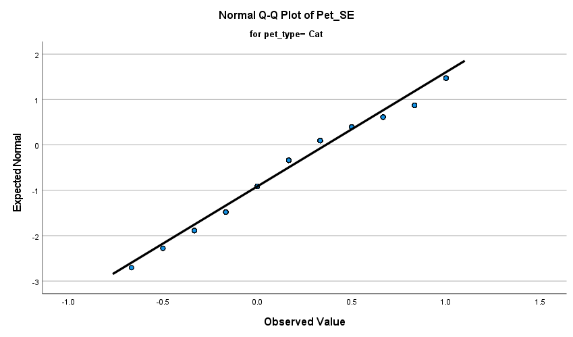 |
| Tradition | | | |
| 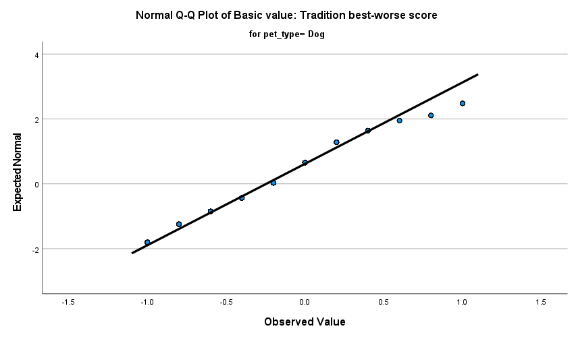 | 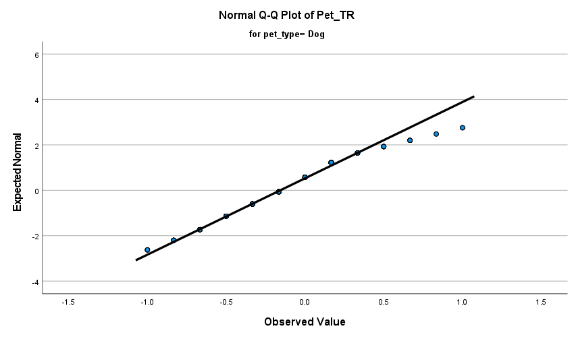 | 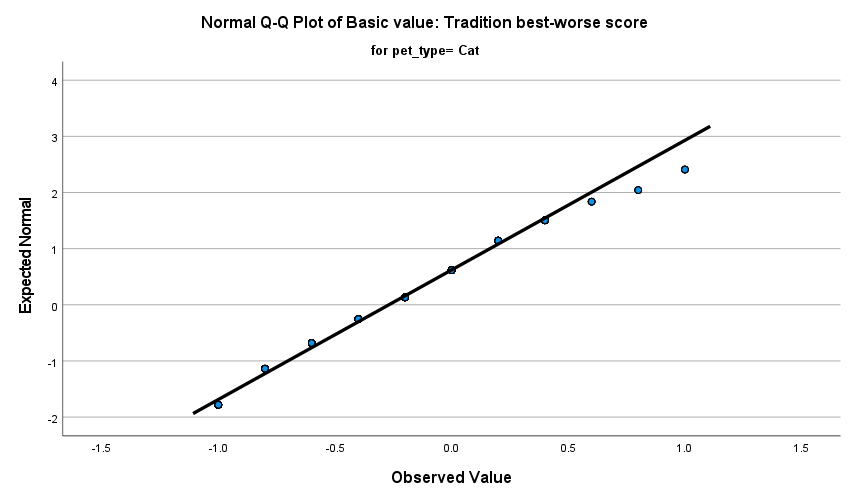 | 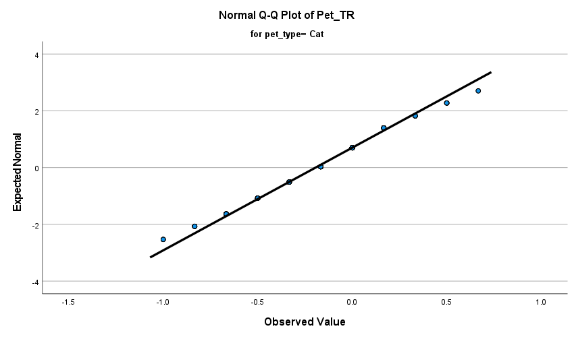 |
| Conformity | | | |
| 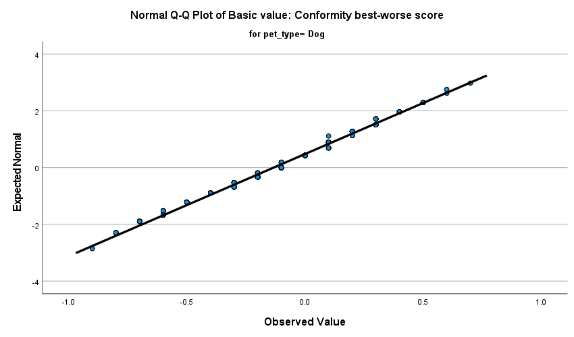 | 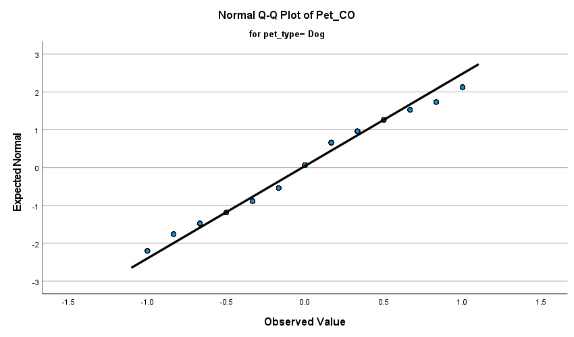 | 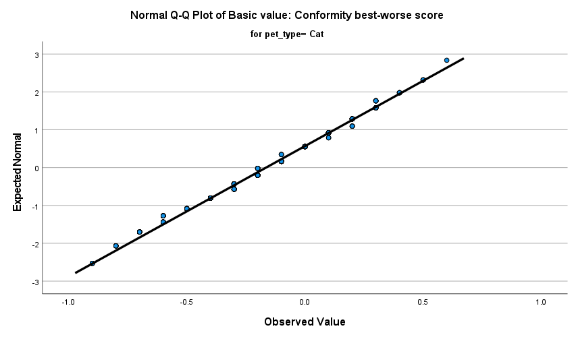 | 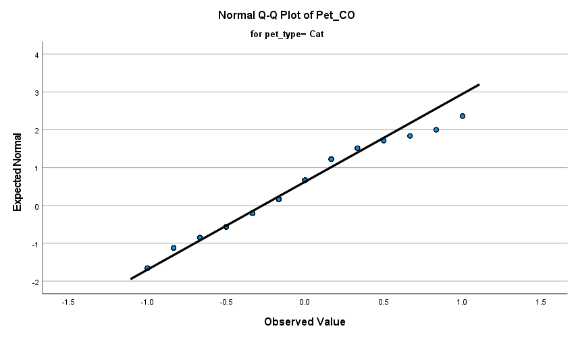 |
| Benevolence | | | |
| 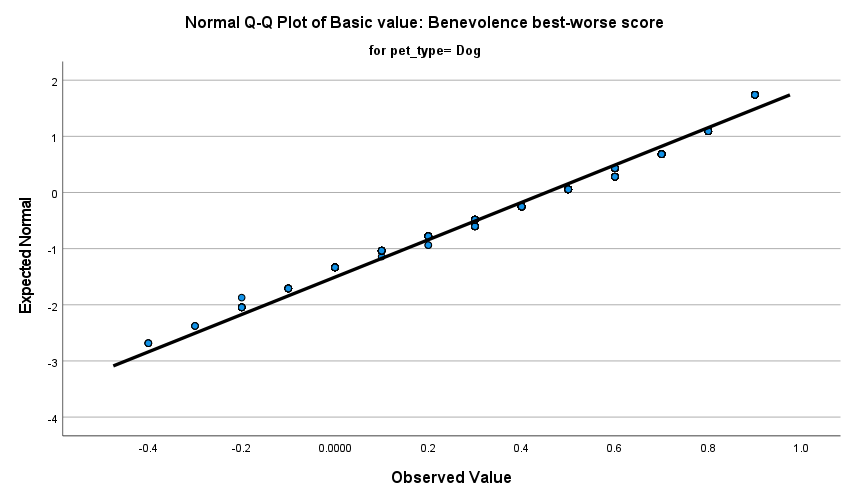 | 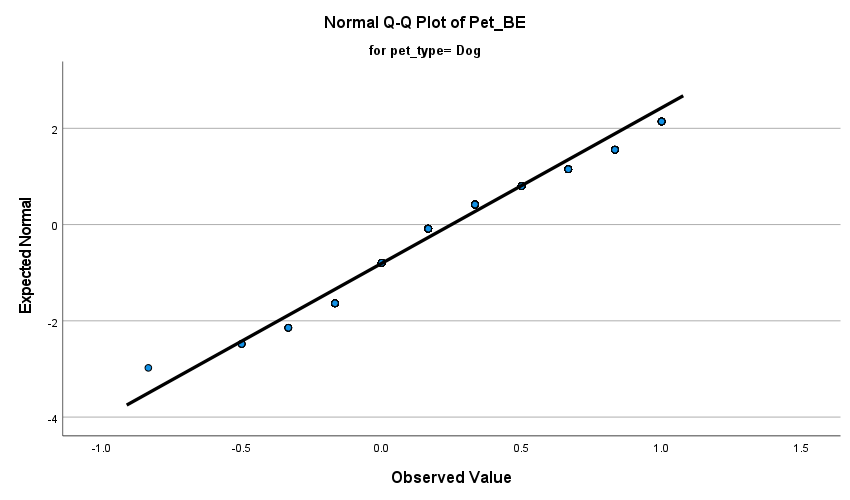 | 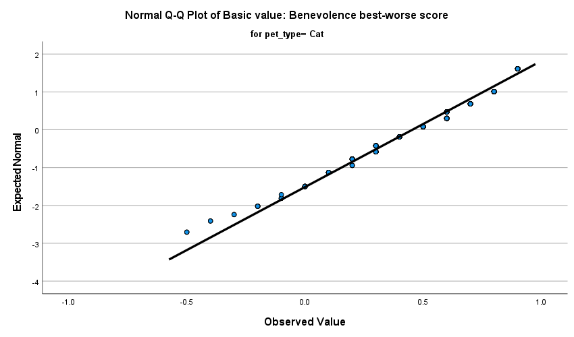 | 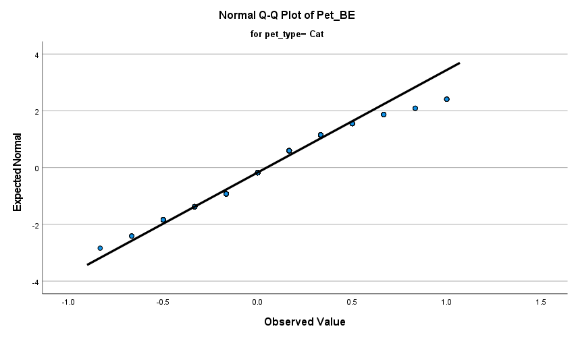 |
| Universalism | | | |
| 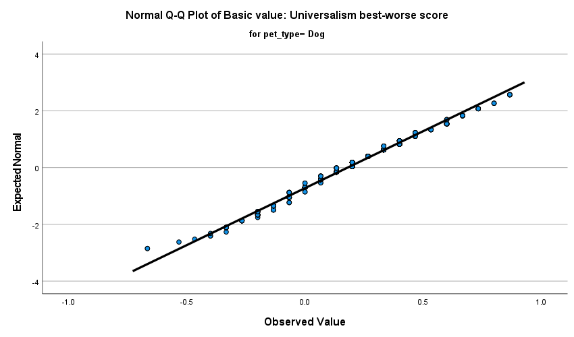 | 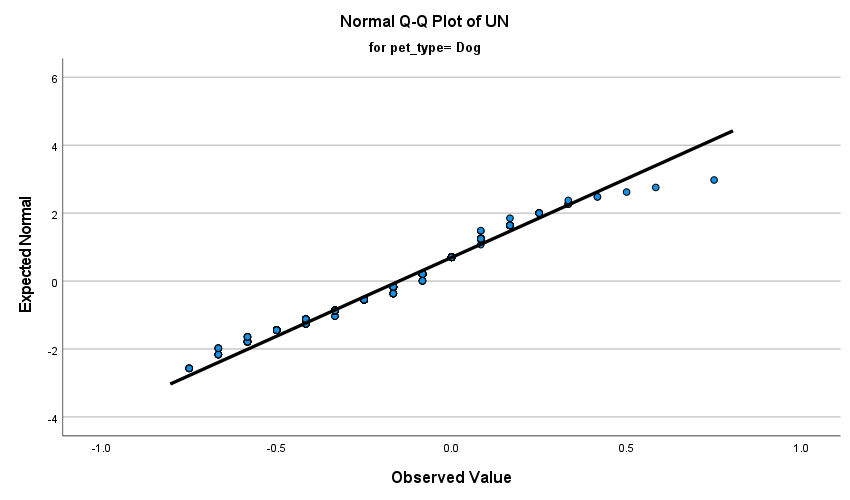 | 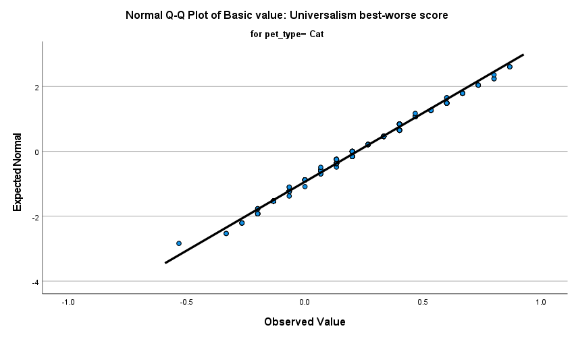 | 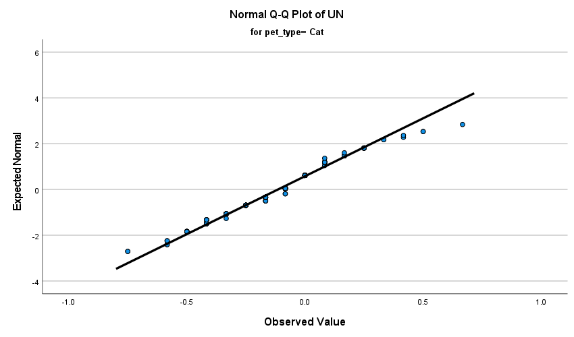 |
| Life Satisfaction | | | |
| 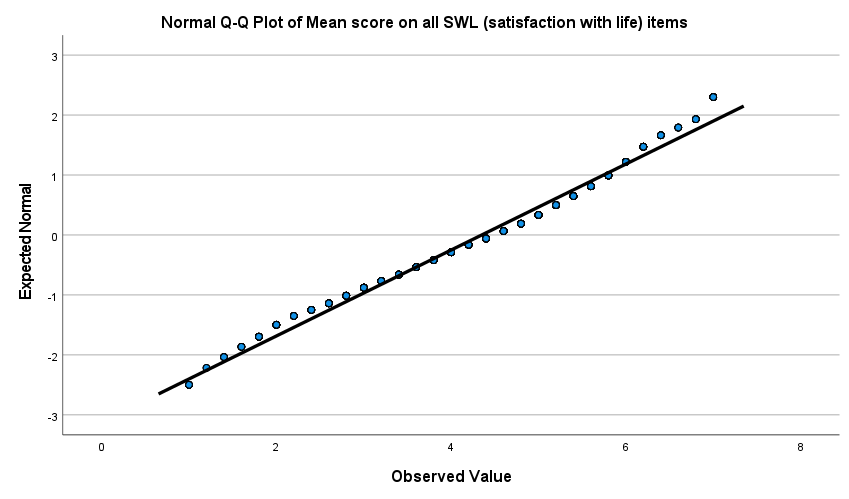 | | | |

*Note*. Dog owners and dogs values *n* = 684, Cat owners and cats values *n* = 438, Life Satisfaction *N* = 1,122.

**Supplementary Table 5.** Means and Mean Differences in Values Scores for Dog and Cat Owners.

| Values | Dog Owners | | Cat Owners | | *t* | *p* | Cohen’s *d* |
| --- | --- | --- | --- | --- | --- | --- | --- |
|  | *M* | *SD* | *M* | *SD* |  |  |  |
| Self-direction | 0.08 | 0.26 | 0.12 | 0.27 | -2.05 | .041 | -.125 |
| Stimulation | 0.01 | 0.35 | -0.02 | 0.35 | 1.43 | .154 | .087 |
| Hedonism | 0.12 | 0.37 | 0.09 | 0.36 | 1.12 | .262 | .069 |
| Achievement | -0.32 | 0.43 | -0.37 | 0.43 | 2.02 | .044 | .124 |
| Power | -0.52 | 0.34 | -0.54 | 0.32 | 1.26 | .210 | .077 |
| Security | 0.31 | 0.26 | 0.33 | 0.26 | -1.21 | .228 | -.074 |
| Tradition | -0.25 | 0.40 | -0.27 | 0.43 | 0.91 | .361 | .057 |
| Conformity | -0.13 | 0.28 | -0.17 | 0.29 | 1.85 | .065 | .113 |
| Benevolence | 0.45 | 0.30 | 0.45 | 0.30 | -0.09 | .926 | -.006 |
| Universalism | 0.18 | 0.25 | 0.22 | 0.24 | -2.70 | .007 | -.165 |

*Note.* Dog owners and dogs *n* = 684, cat owners and cats *n* = 438. All comparisons were two-tailed, independent samples *t*-tests (*df* = 1120, except Tradition *df* = 874.36).

**Supplementary Table 6.** Correlations Between Basic Values and Life Satisfaction.

|  | Life Satisfaction | | | |
| --- | --- | --- | --- | --- |
|  | Dog Owners | Dogs | Cat Owners | Cats |
| Self-direction | -.07 | .01 | -.03 | .02 |
| Stimulation | .01 | -.03 | -.07 | -.00 |
| Hedonism | .10^*^ | -.07 | .04 | .03 |
| Achievement | .04 | .00 | -.06 | -.02 |
| Power | -.04 | .02 | -.03 | .04 |
| Security | -.00 | -.05 | -.01 | -.04 |
| Tradition | .14^***^ | .05 | .03 | -.03 |
| Conformity | .08^*^ | .10^**^ | .06 | -.00 |
| Benevolence | -.01 | -.03 | .06 | -.01 |
| Universalism | -.03 | .01 | -.00 | -.02 |

*Note.* Spearman’s rho. Dog Owners and Dogs *n* = 684, Cat Owners and Cats *n* = 438; ^*^ *p* < .05, ^**^ *p* < .01, ^***^ *p* < .001.

**Supplementary Table 7.** Main Effects and the Interaction Effect of Pet Type and Gender on Life Satisfaction.

| Source | *SS* | *df* | *MS* | *F* | *p* | η_p_^2^ |
| --- | --- | --- | --- | --- | --- | --- |
| Corrected Model | 19.635^a^ | 4 | 4.909 | 2.536 | .039 | .009 |
| Intercept | 1838.019 | 1 | 1838.019 | 949.725 | <.001 | .460 |
| Age | .014 | 1 | .014 | .007 | .933 | .000 |
| Pet Type | 8.072 | 1 | 8.072 | 4.171 | .041 | .004 |
| Gender | 7.217 | 1 | 7.217 | 3.729 | .054 | .003 |
| Pet Type * Gender | .011 | 1 | .011 | .006 | .940 | .000 |
| Error | 2161.749 | 1117 | 1.935 |  |  |  |
| Total | 23404.600 | 1122 |  |  |  |  |
| Corrected Total | 2181.384 | 1121 |  |  |  |  |

*Note*. R Squared = .010 (Adjusted R Squared = .005); Dependent variable: life satisfaction; *SS* = Type III Sum of Squares; *df* = degrees of freedom; *MS* = mean square; η_p_^2^ = partial eta squared.

**Supplementary Table 8.** Polynomial Regression and Response Surface Results by Pet Type and Gender.

| Pet type and gender | Values | Estimated regression model | | | | | | |  | | |  | | Surface Parameters | | | |
| --- | --- | --- | --- | --- | --- | --- | --- | --- | --- | --- | --- | --- | --- | --- | --- | --- | --- |
|  |  | *b_0_* | *b_1_* | *b_2_* | *b_3_* | *b_4_* | *b_5_* | *p*_10_ | | *p*_11_ | *p*_20_ | | *p*_21_ | *a*_1_ | *a*_2_ | *a*_3_ | *a*_4_ |
| All Dog Owners | HE | 4.56^*^ | 0.10 | -0.54 | -0.05 | 0.78 | 0.14 | -0.94 | | 1.28 |  | |  | -0.44 | 0.86 | 0.64 | -0.69 |
|  | ACH | 4.55^*^ | -0.13 | -0.16 | -0.56^*^ | -0.13 | -0.18 | -0.84 | | -6.27 |  | |  | -0.29 | -0.86 | 0.03 | -0.61 |
| Male Dog Owners | HE | 4.72^*^ | -0.34 | -0.54 | -0.55 | 1.11 | 0.32 | 0.10 | | 2.06 |  | |  | -0.88 | 0.88 | 0.20 | -1.33 |
|  | PO | 4.65^*^ | 0.33 | 0.64 | -0.22 | 2.31^*^ | -0.49 | 0.11 | | 0.89^*^ |  | |  | 0.97 | 1.60 | -0.30 | -3.02^*^ |
| Female Dog Owners | HE | 4.43^*^ | 0.85^*^ | -0.61 | -0.10 | -0.00 | 0.28 | 825.73 | | -187.34 |  | |  | 0.24 | 0.18 | 1.47^*^ | 0.19 |
|  | BEN | 4.38^*^ | -0.44 | -0.18 | 0.87 | -1.11 | 0.76 |  | |  | -0.11 | | 1.10 | -0.62 | 0.51 | -0.25 | 2.74 |
| Female Cat Owners | SD | 4.03^*^ | -0.39 | -0.22 | 1.24 | -0.27 | 0.80 |  | |  | -0.46 | | 3.63 | -0.61 | 1.78 | -0.16 | 2.31 |
|  | BEN | 3.86^*^ | 0.37 | 0.66 | 0.28 | -1.96^*^ | 0.67 |  | |  | -0.12 | | 0.82^*^ | 1.03 | -1.01 | -0.28 | 2.91^*^ |

*Note.* Dog owners *n* = 684, male dog owners *n* = 266, female dog owners *n* = 418; Cat owners *n* = 438, male cat owners *n* = 126, female cat owners *n* = 312; ^*^ *p* < .05. HE = hedonism, ACH = achievement, PO = power, BEN = benevolence, SD = self-direction.
